# Recent advances in bio-inspired ionic liquid-based interfacial materials from preparation to application

**DOI:** 10.3389/fbioe.2023.1117944

**Published:** 2023-01-19

**Authors:** Zhe Zhang, Ran Zhao, Shutao Wang, Jingxin Meng

**Affiliations:** ^1^ CAS Key Laboratory of Bio-inspired Materials and Interfacial Science, Technical Institute of Physics and Chemistry, Chinese Academy of Sciences, Beijing, China; ^2^ University of Chinese Academy of Sciences, Beijing, China; ^3^ Qingdao Casfuture Research Institute Co., Ltd., Qingdao, China; ^4^ Binzhou Institute of Technology, Binzhou, China

**Keywords:** bio-inspired, ionic liquid, interfacial, adhesion, regulation

## Abstract

Natural creatures always display unique and charming functions, such as the adhesion of mussels and the lubrication of Nepenthes, to maintain their life activities. Bio-inspired interfacial materials infused with liquid, especially for ionic liquids (ILs), have been designed and prepared to meet the emerging and rising needs of human beings. In this review, we first summarize the recent development of bio-inspired IL-based interfacial materials (BILIMs), ranging from the synthesis strategy to the design principle. Then, we discuss the advanced applications of BILIMs from anti-adhesive aspects (e.g., anti-biofouling, anti-liquid fouling, and anti-solid fouling) to adhesive aspects (e.g., biological sensor, adhesive tape, and wound dressing). Finally, the current limitations and future prospects of BILIMs are provided to feed the actual needs.

## 1 Introduction

In nature, the structure and function of biotas provide continuous inspiration for technological development and material fabrication. Learning from nature, a series of bio-inspired interfacial materials have been developed for meeting actual needs, such as lotus leaf-inspired self-cleaning materials (Feng et al., 2002), cactus-inspired water-collecting materials (Ju et al., 2012), sharklet skin-inspired drag-reducing materials (Bechert et al., 1997), and polar bear hair-inspired thermal insulation materials (Chen et al., 2016; Cui et al., 2018). The scientific studies of bio-inspired interfacial materials provide new insights into crucial fields, including energy conservation, environment protection, and information transfer.

Inspired by pitcher plants, a liquid-infused material was developed for antifouling and anti-corrosion by preventing direct contact between fouling and the substrate through the liquid barrier layer on the surface. Ionic liquid (IL) is a liquid organic salt composed of organic cations and inorganic or organic anions. In the 1970s and 1980s, the first generation of ILs with non-volatility and thermal stability was established. To meet practical needs, the second generation of ILs with adjusted and specific physicochemical properties was developed in 1992. In the early 21st century, the third generation of biocompatible ILs attracted more research interest. The fourth generation of ILs with unique and unpredictable properties in solution or after mixing with other molecular liquids was first proposed in 2018. As a non-volatile lubricant, IL has attracted significant attention for extending the durability of liquid-infused surfaces. In contrast, the IL-based interfacial materials exhibit strong adhesion when the IL is tightly bonded within the polymer network. Meanwhile, the lubricity and adhesion of IL-based interfacial materials can be controlled intelligently by regulating the state of IL on the surface or inside the polymer. Therefore, the potential applications of advanced IL-based interfacial materials can be improved and enriched by employing appropriate ILs because of their attractive advantages such as adjustable wettability, structural variability, and desirable electrochemical stability.

**FIGURE 1:**
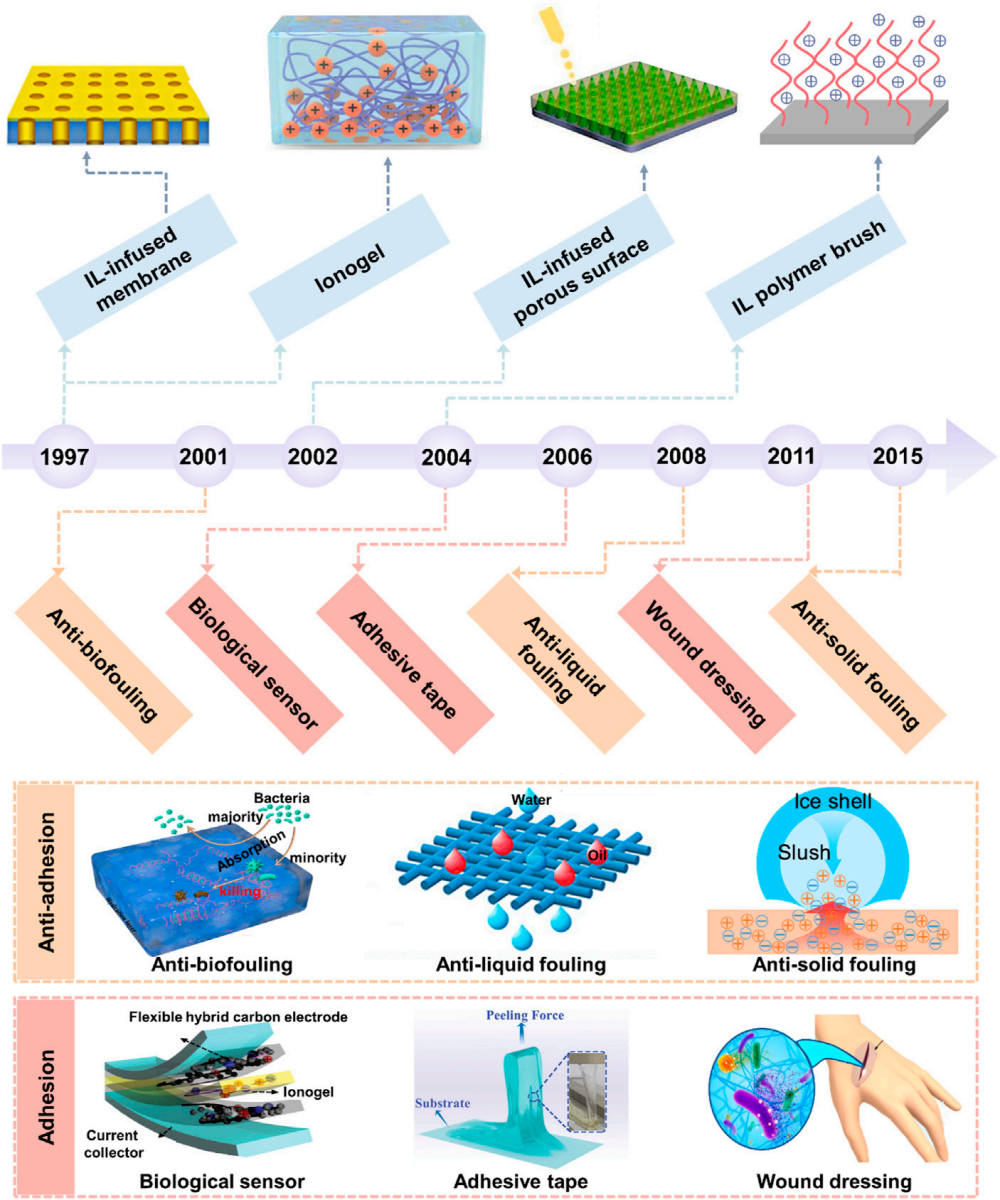
Milestones of the development and application of BILIM. The development of ILs has gone through three generations ranging from the first generation of unstable ILs and the second generation of stable ILs to the third generation of functional ILs. There are four main forms of IL-based interfacial materials, including 1D IL polymer brush, 2D IL-infused porous surface and supported membrane, and 3D ionogel. The applications of BILIMs are mainly divided into anti-adhesion (anti-biofouling, anti-scaling, and anti-icing) and adhesion (biological sensor, adhesive tape, and wound dressing). Reproduced with permission (Zhang et al., 2019). Copyright 2019, American Chemical Society. Reproduced with permission (Zhuo et al., 2020). Copyright 2020, American Chemical Society. Reproduced with permission (Deng et al., 2020). Copyright 2020, American Chemical Society. Reproduced with permission (Cho et al., 2020). Copyright 2020, WILEY-VCH Verlag GmbH & Co. KGaA, Weinheim. Reproduced with permission (Yao et al., 2022). Copyright 2022, Wiley-VCH GmbH. Reproduced with permission (Wang et al., 2020). Copyright 2020, American Chemical Society.

Distinct from the previous reviews on IL materials, this review mainly reexamines the development history of bio-inspired IL-based interfacial materials (BILIMs), as shown in Figure 1. First, we summarize the synthesis strategy and design principle of BILIMs. Then, we introduce the recent advances of BILIM applications from anti-adhesion (e.g., anti-biofouling, anti-liquid fouling, and anti-solid fouling) to adhesion (e.g., biological sensor, adhesive tape, and wound dressing). Finally, we discuss the limitation and future development of BILIM applications.

## 2 Preparation method of BILIMs

Inspired by nature, four main types of BILIMs divided by spatial dimension have been prepared, ranging from one-dimensional (1D) IL polymer brush, 2D IL-infused porous surface, and supported membrane to 3D ionogel (Figure 1).

### 2.1 IL polymer brush

By linking one end of the IL polymer chain, a 1D IL polymer brush can be formed on the surface of various substrates, thereby integrating excellent chemical and physical properties of ILs into these substrates (Washiro et al., 2004; Laloyaux et al., 2010; Buddingh et al., 2021). However, traditional free radical polymerization meets with the difficulty of controlling the polymer structure and molecular weight. To overcome this problem, four main methods with controlled radical polymerization are introduced in brief as follows: atom transfer radical polymerization (ATRP), reversible addition-fragmentation chain-transfer (RAFT), nitroxide-mediated radical polymerization (NMP), and ring-opening metathesis polymerization (ROMP). These methods rely on the reversible storage of active radicals to reduce termination events relative to the total number of polymer chains. Thus, this allows for better control not only of molecular weight and dispersion but also of the end composition of the chain, allowing the construction of a specialized graft structure of the chain.

**FIGURE 2:**
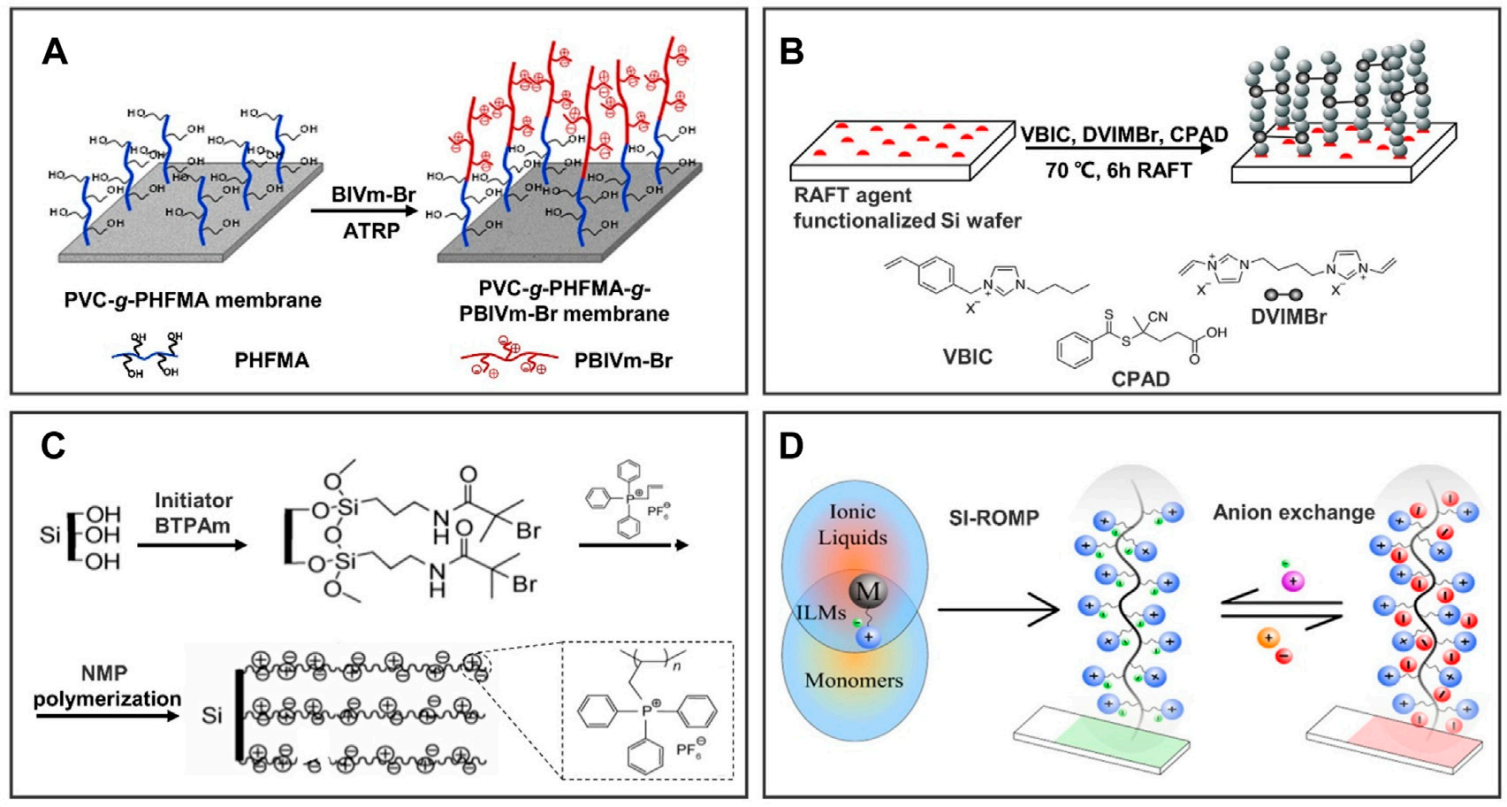
Preparation methods of 1D IL polymer brushes by chemical reaction. Numerous studies have reported on the preparation methods of ILs polymer brushes; four general preparation methods are introduced in brief as follows: **(A)** atom transfer radical polymerization (ATRP); **(B)** reversible addition-fragmentation chain transfer (RAFT); **(C)** ring-opening metathesis polymerization (ROMP); and **(D)** nitroxide-mediated radical polymerization (NMP). **(A)** reproduced with permission (Cheng et al., 2018). Copyright 2017, John Wiley and Sons, Ltd; **(B)** reproduced with permission (Demirci et al., 2020). Copyright 2020, American Chemical Society; **(C)** reprinted with permission (Njoroge et al., 2017). Copyright 2017, American Chemical Society; **(D)** reproduced with permission (Yang et al., 2010). Copyright 2010, Science China Press and Springer-Verlag Berlin Heidelberg.

Generally, the IL polymer brush was prepared on the surface of substrates by forming active free radicals and then polymerization such as ATRP, RAFT, and NMP. ATRP was performed by utilizing organic halides as initiators and transition metal complexes as halogen atom carriers to form a fast conversion “free ion to silyl ketene acetal” equilibrium system through a redox reaction, thus realizing the control of polymerization reaction. ATRP has been widely used in the preparation of modified IL membranes due to its strong controllability. For example, Figure 2A shows that Cheng et al. (2018) grafted poly(1-butyl-3-vinylimidazolium bromide) (PBVIm-Br) onto the surface of poly(vinyl chloride) (PVC) membrane by ATRP. The hydrophilicity of the poly(ionic liquid) (PIL) polymer brush-modified membrane is significantly enhanced by positive charge, which has a potential application in membrane separation. In addition, a special chain transfer agent with a high chain transfer constant is added to the polymerization system, which enables the degenerate transfer between the growing free radical and the chain transfer agent, reduces the concentration of free radicals, narrows the molecular weight distribution, and enables the polymerization to reflect the controllable/“active” characteristics. This reaction process is called RAFT, which has a promising application because of its wide range of monomers. However, RAFT also has many shortcomings, such as a complex preparation process, difficulty obtaining commercial reagents, easily caused chain termination, and other problems. To obtain the homogeneous brush layer on the substrate, as shown in Figure 2B, 1,4-di(3-vinylimidazolium)butane dibromide (DVIMBr) as the crosslinker was used to graft 1-vinylbenzyl-3-butylimidazolium bromide (VBIC) onto the membrane through RAFT (Demirci et al., 2020). Additionally, Figure 2C shows that a PIL-grafted silicon surface was prepared by NMP, and the wettability of the surface can be changed from hydrophilicity to hydrophobicity by changing the anion type through ion exchange (Yang et al., 2010). In essence, NMP generates free radicals from nitrogen–oxygen compounds and forms dormant species of monomer free radicals in the process of polymerization, thereby achieving controlled polymerization. Although this method has the advantages of being environmentally friendly and simple, it remains a challenge in scalable applications.

Different from the abovementioned methods, the double bond contained in the monomer is still retained in the polymer obtained by ROMP. ROMP is the ring-opening polymerization of ring alkenes. Its occurrence requires the following four aspects: the presence of ring alkenes, carbene complex catalysts, the breaking of double bonds, and the end-to-end connection. For instance, Njoroge et al. (2017) grafted 3-[(bicyclo[2.2.1]hept-5-en-2-yl)methyl]-1,2-dimethylimidazol-3-ium hexafluorophosphate ([N_1_-dMIm][PF_6_] to Au or silicon substrates through ROMP as shown in Figure 2D. As a result of the simple and efficient preparation process, ROMP has been widely used for preparing an IL polymer brush-modified surface.

Although IL polymer brushes strengthen the interfacial interaction between ILs and substrate, it still confronts some problems such as the uncontrollable accurate density of grafted ILs and the limited types of available ILs (Men et al., 2013). At present, IL brushes are basically grafted onto the substrate by a polymerization reaction.

### 2.2 IL-infused porous surface

To solve the fouling problem of pollutants, the slippery liquid-infused porous surface (SLIPS) has been developed by Aizenberg due to the existence of the lubricant layer. However, the SLIPS often face a problem caused by the loss of lubricant from the coating surface, and durability is still a challenge for the SLIPS. The loss of lubricant is a common phenomenon that may lead to the failure of SLIPS coating. Due to their advantages (e.g., adjusted surface energy and negligible vapor pressure), ILs as one of the lubricants may alleviate this failure. The IL-infused porous surface was prepared by superwetting of the ILs on the surface through the capillary interaction or the hydrophobic interaction between the micro-/nanostructure and the ILs (Liang et al., 2002; Salbaum et al., 2021). To obtain excellent antifouling properties, porous surfaces can be prepared by many promising methods, such as the hydrothermal method, freeze–drying, spraying, and layer-by-layer suction flow methods.

**FIGURE 3:**
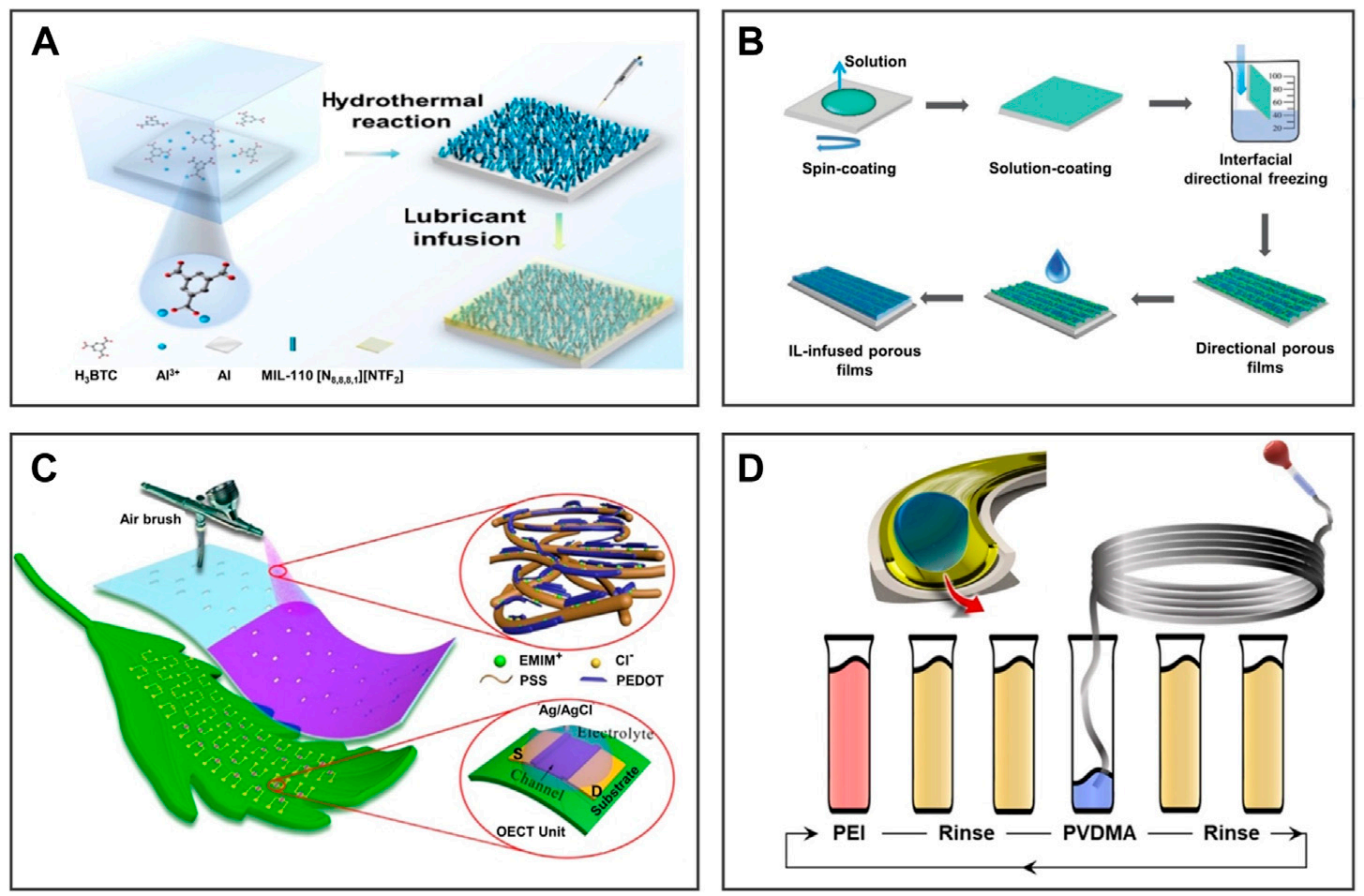
Preparation methods of 2D IL-infused porous surfaces. Schematic diagram of the IL-infused porous surface obtained *via* the hydrothermal method, freeze–drying, spraying, and layer-by-layer method. **(A)** The hydrothermal method is a process of precursor chemical reaction on the substrate under high temperature and high-pressure conditions. **(B)** The interfacial directional freezing technique is usually used to prepare porous materials with anisotropic interfaces. **(C)** Spraying–deposition is a process of spraying solution to the substrate surface through a spray gun to obtain coating. **(D)** The layer-by-layer suction flow method is suitable for a pipe, which uses an external injection apparatus to slowly and evenly infuse solution into the pipe. **(A)** is reproduced with permission (Li et al., 2022). Copyright 2021, Elsevier Inc.; **(B)** reproduced with permission (Wang Z. et al., 2018). Copyright 2018, WILEY-VCH Verlag GmbH and Co. KGaA, Weinheim; **(C)** reprinted with permission (Wu et al., 2020). Copyright 2020, American Chemical Society; **(D)** reproduced with permission (Agarwal et al., 2021). Copyright 2021, American Chemical Society.

The hydrothermal method is a process of precursor chemical reaction on the substrate under high-temperature and high-pressure conditions. For example, Li et al. (2022) prepared a MOF-based surfactant with dual functions of contact killing and fouling release on an aluminum sheet by the one-step hydrothermal reaction as shown in Figure 3A. IL-[N_8,8,8,1_][NTf_2_] was captured by the Al-MIL-110 porous surface to obtain an anti-biofouling coating with excellent stability and low lipopolysaccharide adsorption capacity. It as a novel synergistic coating has potential applications for the degradation and release of pollutants. Compared with the traditional *in situ* synthesis method, the hydrothermal method only needs to place the substrate directly into the precursor solution to obtain a porous surface. However, the hydrothermal method also has some obvious disadvantages, such as the need for high temperature and high pressure and other harsh conditions, which makes it more dependent on production equipment, which also hinders the universality of preparing porous surfaces on any substrate. Moreover, the mechanical robust and long-term properties of this coating are still a challenge in practical application.

The interfacial directional freezing technique is usually used to prepare porous materials with anisotropic interfaces. For instance, on the basis of the anisotropic porous film, Figure 3B shows that Wang X. et al. (2018)prepared the smooth surface with a photoelectric synergistic response using the interfacial directional freezing technology of the poly(3-hexylthiophene-2,5-diyl)/[6,6]-phenyl-C_61_-butyric acid methyl ester (P3HT/PCBM) binary system. Compared to the porous material prepared by the traditional directional freezing technology, the porous material prepared by the interface directional freezing technology exhibits anisotropy from bulk to the interface. However, this method is expensive and difficult to scalable manufacturing.

Spraying–deposition is a process of spraying solution to the substrate surface through a spray gun to obtain coating. As shown in Figure 3C, Wu et al. (2020) demonstrated a simple spray–deposition method to prepare an organic electrochemical transistor (OECT) made of IL-doped poly(3,4-ethylenedioxythiophene): poly(styrene sulfonate) (PEDOT: PSS). The spray–deposition technology provides a convenient way to prepare high-performance OECT channels with scalable manufacturing, excellent stability, high yield, and low cost. However, spraying–deposition is difficult to coat evenly when it comes to the inner surface of objects with a narrow inner cavity, such as the pipe wall with a small inner diameter. The layer-by-layer suction flow method remedies this deficiency, which uses an external injection apparatus to slowly and evenly infuse solution into the pipe. For instance, Agarwal et al. (2021) produced a uniform smooth coating on the inner surface of narrow pipes as shown in Figure 3D. The coating can be applied to pipes of any size and length for inhibiting the blockage of the pipe. This method provides an opportunity to manufacture coating on the inner surface with various shapes.

These methods merely provide porous surfaces, but the successful preparation of IL-infused surfaces requires suitable interactions between the IL and the porous surface. Also, the function of the IL-infused porous surface depends on the characteristics of the IL existing on the surface, such as repellency, lubricity, wetting properties, and self-healing. Although many efforts have been made to improve the affinity between ILs and porous surfaces, the attractive force is still weak, and the content of ILs trapped by micro-/nanostructures is limited, leading to poor mechanical stability and easy to fail in extreme conditions (Howell et al., 2018). At present, the IL-infused surface still has the dilemma of failure after the loss of surface lubricant.

### 2.3 IL-infused supported membrane

The IL-based supported membrane was prepared by loading ILs into membrane internal channels (Carlin and Fuller, 1997; Branco et al., 2002; Noble et al., 2011; Sasikumar et al., 2018). Compared with traditional supported liquid membranes, ILs with high viscosity can enhance the capillary force between ILs and the supported membrane for improving stability (Sprugis et al., 2019). At present, the reported methods for preparing the IL-infused supported membrane mainly include the impregnation method, pressure difference method, reducing viscosity by heating, and reducing viscosity by solvent.

**FIGURE 4:**
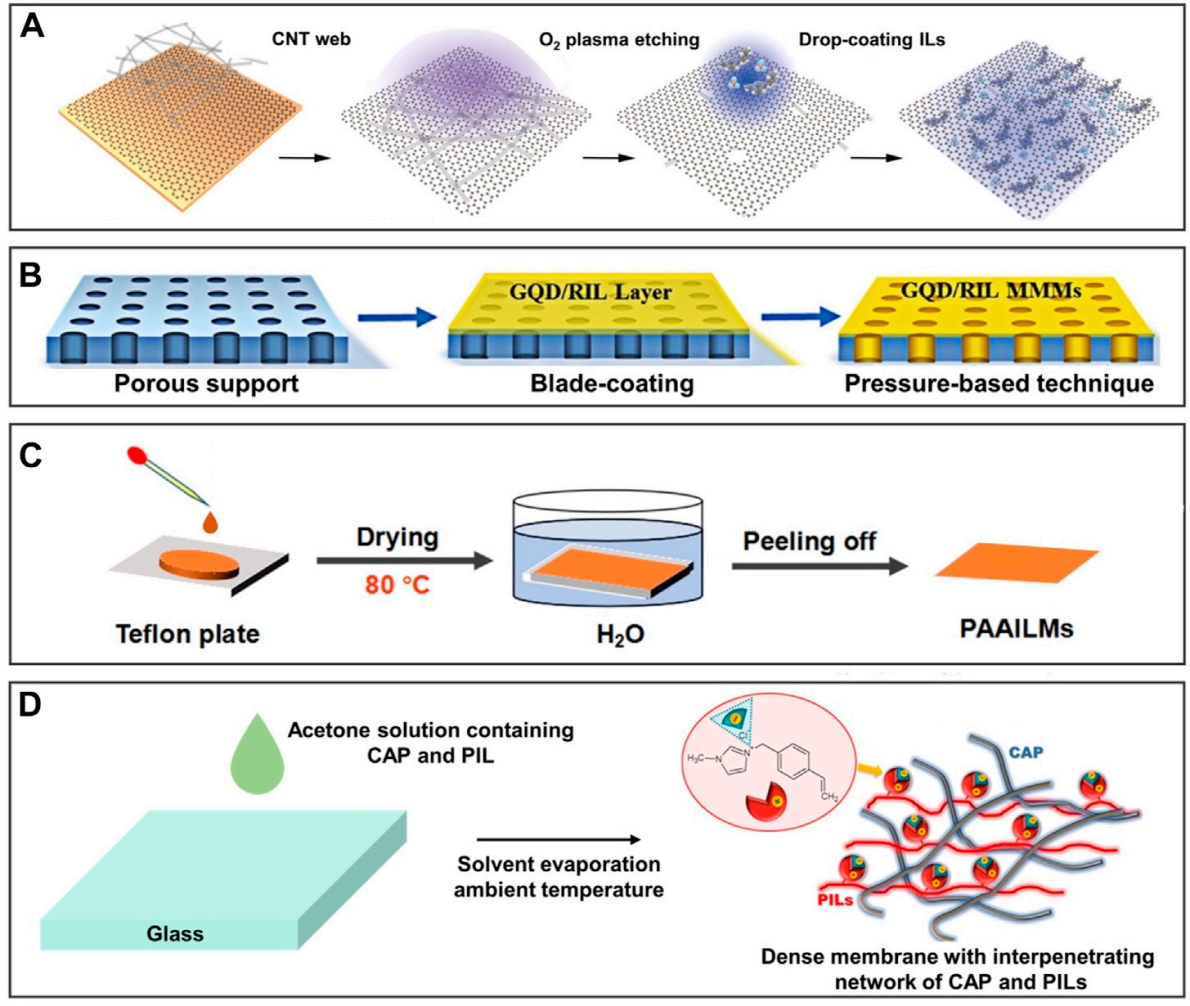
Preparation methods of 2D IL-infused membranes. Schematic diagram of IL polymer brushes grafted surfaces. **(A)** Impregnating ILs into the pores of the graphene-based membrane by capillary force for separating CO_2_ and N_2_. **(B)** Impregnating ILs into the membrane by pressure difference and capillary force for separating ethylene/ethane. **(C)** Reducing the viscosity of PAAILs by heating at 80 °C to impregnate the PVDF-based membrane for separation of amino acids. **(D)** Reducing the viscosity of impregnating 1-methyl-3-(4-vinylbenzyl)-1H-imidazol-3-ium chloride by dissolving in solvent to impregnate the membrane based on cellulose acetate propionate for dehydration of propan-2-ol. **(A)** is reproduced with permission (Guo et al., 2020). Copyright 2020, American Chemical Society; **(C)** reprinted with permission (Dou et al., 2021). Copyright 2020, Wiley-VCH GmbH; **(B)** reproduced with permission (Liu L. et al., 2021). Copyright 2021 Elsevier Inc; **(D)** reproduced with permission (Rynkowska et al., 2017). Copyright 2020, Wiley-VCH GmbH.

The IL-infused supported membrane is usually used to separate mixtures such as CO_2_/N_2_, ethylene/ethane, amino acids, and propan-2-ol/H_2_O. For example, the IL/graphene hybrid membrane was prepared by impregnating ILs into the pores of the film by capillary force (Guo et al., 2020), which can dynamically adjust the chemical affinity of ILs and nanopores for achieving the high permeability of the membrane to separate CO_2_ and N_2_ (Figure 4A). The preparation of this IL-infused supported membrane is simple but limited by the viscosity of ILs and the pore size. ILs impregnate non-uniformly into a large pore size due to weak capillary force, which causes defects and affects the function of the membrane. In addition to the active impregnation method, the passive pressure difference method can also be used to prepare the IL-infused supported membrane. Figure 4B shows that analogous mixed matrix membranes were obtained by the pressure difference method and impregnation method for ethylene/ethane separation (Dou et al., 2021). The non-covalent interactions between ILs and nanofillers induce the arrangement of ILs to form dense mass transfer interface paths for efficient separation. The membrane prepared by this method can overcome the problem of hardly impregnating the channels because of its high viscosity. However, this preparation method is difficult to achieve when the viscosity of ILs is too high.

To solve the problem of high viscosity, raising the temperature or adding solvent seems to achieve reduced viscosity. Mixing poly(amino acid ionic liquids) (PAAILs) with polyvinylidene fluoride (PVDF) and reducing the viscosity of PAAILs by heating at 80°C to prepare the IL-infused supported membrane for separation amino acids in Figure 4C (Liu L. et al., 2021). However, IL-infused supported membranes prepared by increasing temperature and reducing viscosity are limited by the temperature tolerance range of the supported membrane, causing the limited application range. Additionally, the reduced viscosity of ILs by dissolving in solvents allows them to easily penetrate the channel of the supported membrane. As exhibited in Figure 4D, Rynkowska et al. (2017) added 1-methyl-3-(4-vinylbenzyl)-1H-imidazol-3-ium chloride to acetone solution to obtain dense membranes based on cellulose acetate propionate for dehydration of propan-2-ol. This method becomes a promising candidate for preparing IL-infused supported membranes for the advantages of being simple, stable, and uniform.

The multifunctional IL-infused supported membrane can be prepared by employing ILs with tunable properties such as density, viscosity, wettability, and chemical affinity (Hirota et al., 2017). However, huge challenges for the IL-infused supported membrane remained such as the stability under harsh conditions and the preparation on large scale.

### 2.4 Ionogel

Ionogel was formed by infusing ILs into the polymer matrix (Fuller et al., 1997; Lu et al., 2009). In contrast with the IL polymer brush and IL-infused porous surface, the IL content of ionogel increases significantly due to the swelling property of the polymer matrix, which offers significant advantages in terms of durability. Many existing studies have reviewed the preparation methods of ionogels from three main kinds: physical crosslinking method, chemical crosslinking method, and their combinations.

#### 2.4.1 Physical crosslinked ionogel

**FIGURE 5:**
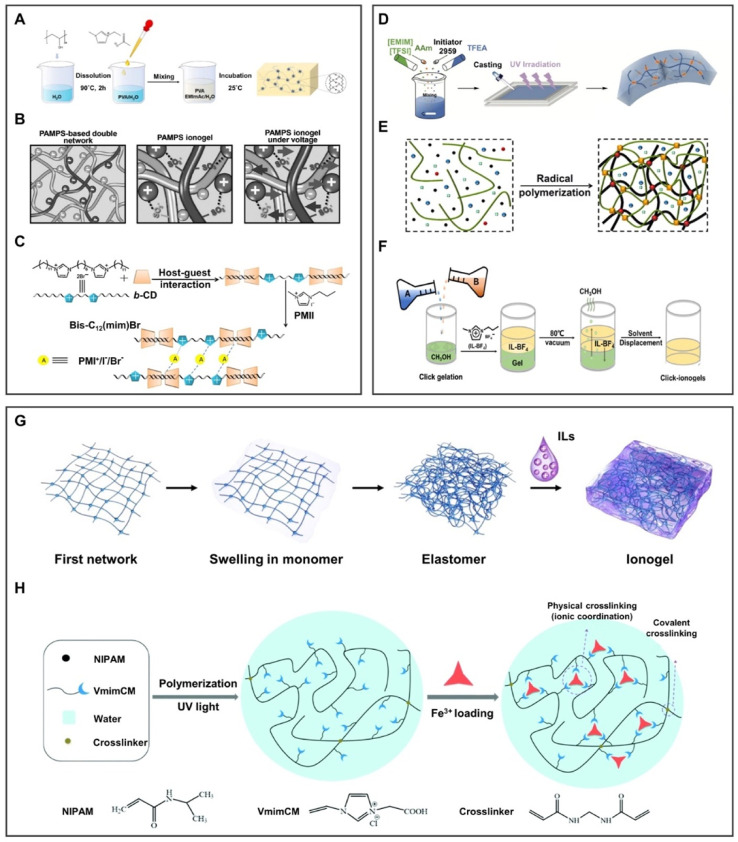
Preparation methods of 3D ionogels. **(A–C)** Schematic diagram of ionogel obtained by physical crosslinking. **(A)** Hydrogen bond was formed by the combination of H atoms and high-electronegative atoms in molecules, endowing ionogel good reversibility and controllable crosslinked strength. **(B)** Electrostatic interaction is chemical bonds formed by the charges interaction between anions and cations. **(C)** Host–guest interaction is the self-assembly of monomer molecules through the recognition of characteristics between the host and the guest. **(A)** is reproduced with permission (Liu Y. et al., 2021). Copyright 2021, American Chemical Society; **(B)** reproduced with permission (Ding et al., 2017). Copyright 2017, WILEY-VCH Verlag GmbH and Co. KGaA, Weinheim; **(C)** reproduced with permission (Zhang et al., 2015). Copyright 2015, Elsevier Ltd. **(D–F)** Schematic diagram of ionogel obtained by chemical crosslinking. **(D)** Photoinitiated polymerization under the participation of ultraviolet light. **(E)** Thermal polymerization under the participation of thermal. **(F)** Click chemistry under the reaction of click-functional groups to form stable conjugates in mild conditions. Image **(D)** is reproduced with permission (Xu et al., 2021). Copyright 2021, Wiley-VCH GmbH; **(E)** reproduced with permission (Lan et al., 2020). Copyright 2020, American Chemical Society; **(F)** reproduced with permission (Ren et al., 2019). Copyright 2019, Science. **(G,H)** Schematic diagram of ionogel obtained by the combination of physical and chemical crosslinking through **(G)** hydrogen bonding and thermal polymerization; **(H)** ionic coordination and photopolymerization. Image **(G)** reproduced with permission (Cao et al., 2020). Copyright 2020, The Royal Society of Chemistry; **(H)** reproduced with permission (Sun et al., 2018). Copyright 2018, The Royal Society of Chemistry.

Ionogel can be mainly formed between the IL and the polymer chain by the physical crosslinking method through hydrogen bonds, electrostatic interaction, and host–guest interaction. The hydrogen bond was formed by the combination of H atoms and high-electronegative atoms in molecules, endowing ionogel with good reversibility and controllable crosslinked strength. For instance, Liu Y. et al. (2021) prepared conductive hydrogel by a simple physical crosslinking method. As shown in Figure 5A, PVA/EMImAc/H_2_O hydrogels exhibit the advantages of robustness, high flexibility, and elasticity under the effect of hydrogen bonds, but it is prone to failure in extreme environments. In order to solve this problem, the electrostatic interaction based on chemical bonds between anions and cations can be further introduced. For instance, Ding et al. (2017) used the electrostatic interaction to lock 1-ethyl-3-methylimidazolium dicyandiamide ([EMIm][DCA]) into poly(2-acrylamide-2-methyl-1-propanesulfonic acid) (PAMPS) in Figure 5B. Moreover, its durability may be solved by the host–guest interaction, which is the self-assembly of monomer molecules through the host–guest recognition characteristics. For instance, Zhang et al. (2015) prepared ionogel in Figure 5C by taking advantage of the host–guest interaction between b-cyclodextrin (b-CD) and bisimidazolium ILs (Bis-C_12_(mim)Br). Under the effect of the host–guest interaction, the ionogel can be used as a quasi-solid electrolyte for dye-sensitized solar cells with excellent long-term stability.

The polymer chain entanglement of physically crosslinked ionogel is instantaneously reversible (Sprugis et al., 2019), but the heat-resistance and solvent-resistance are poor, and the permanent deformation is also serious.

#### 2.4.2 Chemical crosslinked ionogel

The chemical crosslinking method mainly includes photopolymerization crosslinking and thermal polymerization crosslinking. Photopolymerization is a typical free-radical polymerization method by activating monomer molecules with light to conduct chain polymerization. This method has the strength of controllability, low polymerization temperature, and high reaction selectivity. For instance, Xu et al. (2021) prepared a multifunctional ionogel through a one-pot photopolymerization, as shown in Figure 5D. This method requires the participation of ultraviolet light, which is difficult to apply in scenarios without light. Fortunately, thermal polymerization makes up for the defects of photopolymerization. Chain polymerization can be conducted by activating monomer molecules in the way of thermal initiation. As illustrated in Figure 5E, Lan et al. (2020) developed a stretchable transparent dual-network ionogel by a simple one-pot thermal polymerization method. However, both photo-initiated polymerization and thermal-initiated polymerization require the input of external energy, making it difficult to synthesize the desired ionogel in a mild environment. Accordingly, Ran et al. prepared IL-click ionogel under mild conditions based on the ionic crosslinking and covalent crosslinking methods in Figure 5F. Click chemistry is a reaction in which a pair of click-functional groups rapidly and selectively reacts with each other to form stable conjugates under mild conditions. Since the click reaction is highly efficient under mild conditions, ionogels can be prepared by simply mixing PEGDA and poly(1-butyl-3vinyl imidazolium fluoborite) (PIL-BF_4_) methanol solutions without additional oxygen, humidity, or heating condition (Ren et al., 2019).

The chemically crosslinked ionogel contains permanent connections formed by covalent bonds, offering ionogel high strength and controllable crosslinked degree.

#### 2.4.3 Physical–chemical crosslinked ionogel

The physical–chemical crosslinking method combines the reversibility of physical crosslinking with the stability of chemical crosslinking. In Figure 5G, the ionogel was prepared through hydrogen bonds and thermal polymerization, which possess transparent, mechanically robust, and high ionic conductivity (Cao et al., 2020). Sun et al*.* prepared a stimuli-responsive ionogel (Figure 5H), comprising chemically crosslinked polymer poly(*N*-isopropylacrylamide) (PNIPAM), physically crosslinked iron ions, and a carboxyl group. Physical crosslinking formed by ion coordination can improve the mechanical strength of ionogel, making it controllable between the strong coordination of Fe^3+^ and the weak coordination of Fe^2+^. In addition, covalent crosslinked PNIPAM and conductive ILs are used as thermal switches to obtain thermal/redox dual stimuli-responsive ionogel (Sun et al., 2018).

Physically and chemically crosslinked ionogels can make up for the shortcomings of a single physically crosslinked or chemically crosslinked ionogel, but the challenge is the rational design of experiments to determine the key performance of ionogels.

In brief, ILs can be physically trapped into the polymer matrix (Mantravadi et al., 2016; Cao et al., 2017; Singh et al., 2017), the weak interfacial force result in poor mechanical properties, but it has good physical properties such as stretchability and self-healing. In addition, ILs can also react with the polymer matrix and be fixed in the polymer matrix through polymerization (Muldoon and Gordon, 2004; Ohno, 2007; Zgrzeba et al., 2015), which strengthens the mechanical properties of ionogel. Meanwhile, the strong crosslinking prevents losing ILs from the surface to the environment. However, ionogel is usually prepared by free radical polymerization, which is limited by the reaction conditions and the type of polymer matrix and ILs. To design required ionogels, the physical–chemical crosslinking method can endow ionogels with excellent physical properties by controlling the crosslinked degree. In addition, ionogels can reversibly expand and contract under external stimuli (e.g., light and heat), which has great potential in the intelligent control system.

## 3 Principle of stable existence of BILIM

BILIMs were divided into three dimensions. The thin layer of IL forms the 1D coating by intermolecular chemical action between IL grafted onto the substrate, and the 2D coating was manufactured by capturing IL through the capillary action or hydrophobic action formed by micro-nano structures on the substrate surface. The inherent porosity of the 3D polymer network and the swelling nature of the network enable the storage of IL. Due to the wide variety of ILs, the properties of ILs also change with the species, such as from hydrophilic to hydrophobic, so the choice of ILs plays a decisive role in the performance of the three coatings. Based on the stable BILIMs, the solid substrate must preferentially wet the IL and repel ambient fluids; meanwhile, the IL and ambient fluids should not dissolve the substrate (Smith et al., 2013).

To ensure the stability of BILIMs in different fluids, the thermodynamic state of coatings can be determined by the contact angles, spread coefficient, and interfacial tension. First, the superwetting of ILs in the air on the substrate surface can be expressed in the following equation:

$$S_{sl\left(a\right)}=\gamma_{sa}-\gamma_{sl}-\gamma_{la},$$ (1)

where *s*, *a*, and *l* represent the substrate, air, and IL, respectively. If $S_{sl\left(a\right)}$ ≥ 0, the IL-based coating was successfully manufactured by the infusion of IL into the substrate; otherwise, IL would remain at top of the substrate at a certain contact angle. Even if the IL-based coating can be stabilized in the air, there is a new criterion for stability when placed in water:

$$S_{sl\left(w\right)}=\gamma_{sw}-\gamma_{sl}-\gamma_{lw},$$ (2)

where w refers to water. IL was captured by the solid texture without being replaced by water when $S_{sl\left(w\right)}$ ≥ 0. If $S_{sl\left(w\right)}$ < 0, water will extrude the IL into the solid texture. Except for the displacement behavior of water, there is also the behavior of IL in the texture-cloaking water droplets causing IL exhaustion:

$$S_{lw\left(a\right)}=\gamma_{sw}-\gamma_{sl}-\gamma_{lw}.$$ (3)

If $S_{lw\left(a\right)}$ < 0, the IL will cloak the water droplet resting on the IL-based coating, which may cause the loss of ILs in the aqueous environment (Peppou-Chapman et al., 2020). In addition, the IL must be insoluble in the working fluid. Based on the aforementioned requirements, there is no best choice of ILs because of their wide varieties, but the types of ILs should be selected according to the actual needs to achieve an excellent performance of the IL-based coating. In general, low surface energy ILs can be injected into textured surfaces while avoiding miscibility with water. ILs do not evaporate quickly in the air because they have almost no saturated vapor pressure. The interfacial tension in the aforementioned equation can be calculated by the following formula:

$$\gamma_{ij}={\left(\sqrt{{\gamma_{i}}^{LW}}-\sqrt{{\gamma_{j}}^{LW}}\right)}^{2}+2\left(\sqrt{{\gamma_{i}}^{+}{\gamma_{i}}^{-}}+\sqrt{{\gamma_{j}}^{+}{\gamma_{j}}^{-}}-\sqrt{{\gamma_{i}}^{+}{\gamma_{j}}^{-}}-\sqrt{{\gamma_{i}}^{-}{\gamma_{j}}^{+}}\right).$$ (4)

For the surface energy component of the substrate, the surface energy parameters of the standard liquid can be obtained from the previous report (Meng and Wang, 2019), measuring the contact angle of the standard liquid on the solid surface and calculating the surface energy using Lewis acid–base theory.

$$\gamma_{s}={\gamma_{s}}^{LW}+{\gamma_{s}}^{AB},$$ (5)

$${\gamma_{s}}^{AB}=2\sqrt{\left({\gamma_{s}}^{+}{\gamma_{s}}^{-}\right),}$$ (6)

$$\gamma_{L}\left(\cos\theta +1\right)=2\sqrt{{\gamma_{L}}^{LW}{\gamma_{S}}^{LW}}+2\sqrt{{\gamma_{L}}^{+}{\gamma_{S}}^{-}}+2\sqrt{{\gamma_{L}}^{-}{\gamma_{S}}^{+},}$$ (7)

where $\gamma^{AB}$ represents the polar acid–base (AB) component of the surface energy, $\gamma^{+}$ represents Lewis acid parameters of the surface energy, $\gamma^{-}$ represents Lewis base parameters of the surface energy, $\gamma^{LW}$ represents the non-polar Lifshitz–van der Waals (LW) component of the surface energy, and *θ* represents the contact angle formed by the standard liquid on the substrate surface.

## 4 Applications

### 4.1 Anti-adhesive application

Undesired adhesion on the surface may damage the surface function and further lead to surface failure. Pitcher plants utilize their slippery characteristics to capture insects as their main source of nitrogen. Corals secrete natural antibacterial substances to resist the attachment of fouling organisms in the sea (Jin et al., 2022). The mucus on fish scales traps water to resist oil pollution. Learning from nature life, BILIM can be widely used in self-cleaning, marine antifouling, biomedical, and other fields because it can inhibit the adhesion of bacteria, oil, scale, and ice.

#### 4.1.1 Anti-biofouling

In 2016, the World Health Organization published a set of alarming data. Approximately 700,000 deaths are attributable to “superbug” infection every year, and the annual number of deaths may increase to 10 million by 2050. The traditional antibacterial materials have developed drug resistance so the need for an alternative and sufficiently powerful antibacterial material so that bacteria never develop resistance is urgent (Wei et al., 2020). Due to the electrostatic effect on disturbing bacterial membranes, IL-based antibacterial materials have attracted extensive research interest (Zhao et al., 2014). Recently, the antibacterial mechanism and intelligent IL-based antibacterial materials have developed rapidly (Zhao et al., 2015).

**FIGURE 6:**
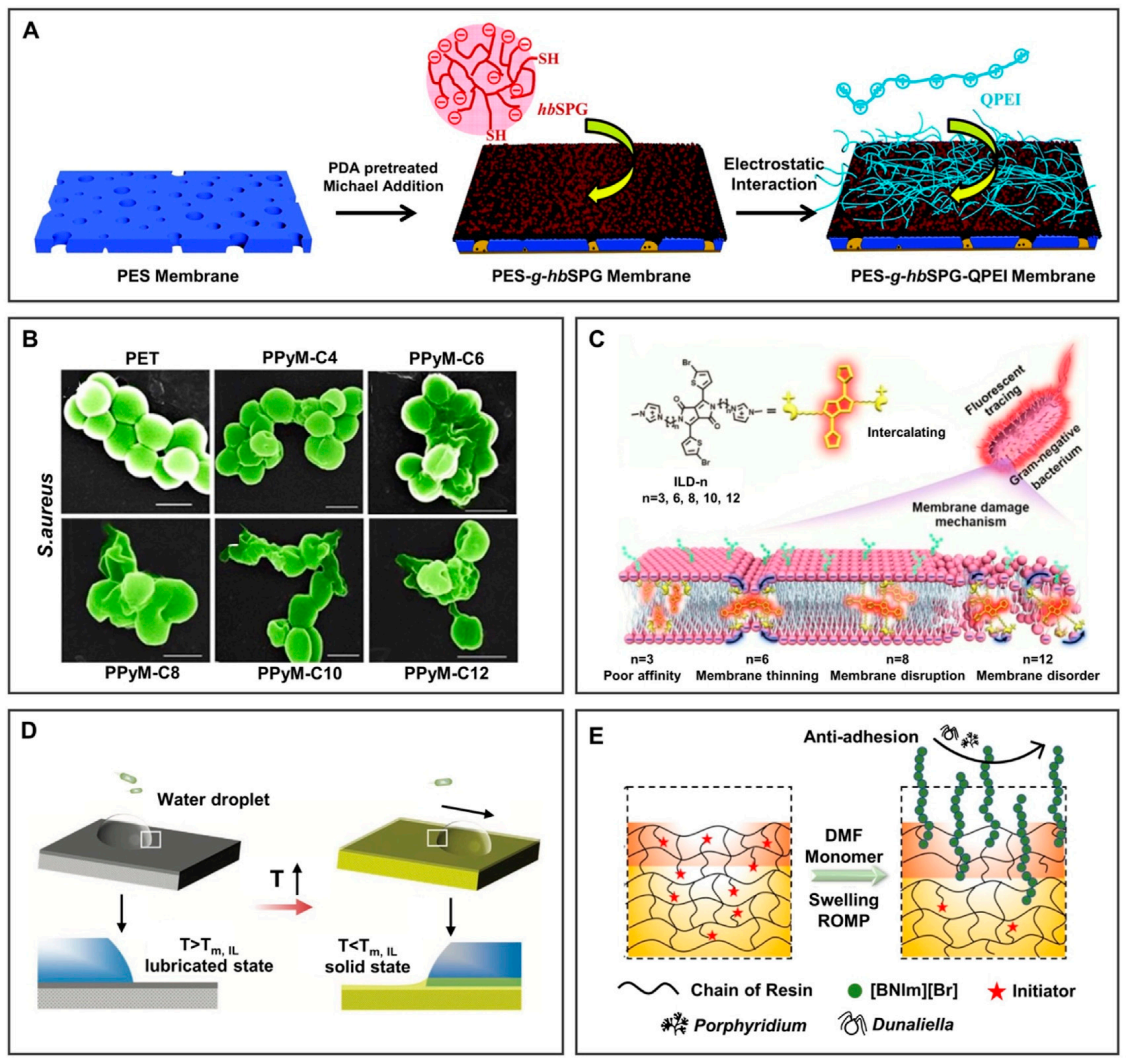
BILIMs for anti-biofouling. **(A)** PILs modified membrane decreased the adsorption of protein obviously after soaking in wastewater for 2 months. Reproduced with permission (Li et al., 2019). Copyright 2019, The Royal Society of Chemistry. **(B)** Antibacterial efficiency of PILs against Gram-positive bacteria increased with the increase of the alkyl chain length of substituents because of hydrophobic segments inserted into bacterial membranes, leading to bacterial death. Reproduced with permission (Qin et al., 2017). Copyright 2017, American Chemical Society. **(C)** The antibacterial efficiency of pyrrolidinium-type IL against *Staphylococcus aureus* increases with the increase of the alkyl chain length of substituents, resulting in instability of the lipid bilayer and further promoting antibacterial activity. Reproduced with permission (Zheng et al., 2020). Copyright 2020, American Chemical Society. **(D)** Responsive self-replenishing ionogel with renewable anti-biofouling properties. Reproduced with permission (Ye et al., 2019). Copyright 2019, WILEY-VCH Verlag GmbH and Co. KGaA, Weinheim. **(E)** Bio-inspired sharklet surface can remove algae and shows good anti-biofouling performance. Reproduced with permission (He et al., 2021). Copyright 2021, Elsevier B.V.

As shown in Figure 6A, the anionic sulfonated hyperbranched polyglycerol (hbSPG) and cationic quaternized polyethylenimine (QPEI) were introduced onto the poly(ether sulfone) (PES) membrane (Li et al., 2019). The membrane showed anti-protein adsorption through the surface hydration network, preventing the adsorption of hydrophobic impurities and antibacterial performance because of its hierarchical architecture and the free quaternary ammonium base after being immersed in wastewater for 2 months, showing great potential in the application of anti-protein pollution.

Moreover, the antibacterial mechanism of BILIMs and the effect of chain length on antibacterial ability were further studied. In Figure 6B, Qin et al. (2017) showed that the antibacterial efficiency of PILs against Gram-positive bacteria increased with the increase of the alkyl chain length of substituents, the reason may be that hydrophobic segments are more easily inserted into bacterial membranes, leading to bacterial death. Furthermore, the antibacterial mechanism was systematically explored at the molecular level (Zheng et al., 2020). As shown in Figure 6C, ILs were readily embedded into the bacterial membrane as the molecular size increases, resulting in the instability of the lipid bilayer and further promoting antibacterial performance. This work demonstrated the electrostatic interactions between molecular sizes of IL-based materials and Gram-negative bacteria with the effect of an antibacterial ability. Moreover, the responsive self-replenishing coating offers an idea strategy for the renewable anti-biofouling surface. In Figure 6D, Ye et al. (2019) mixed the solid/liquid binary mixture of ILs with a semicrystalline polymer. After heating, the surface of the ionogel changes from a solid to a liquid-infused state, which promotes the removal of biofilm/bacteria. After the surface is damaged, ILs released from the inside to the surface and crystallized on the surface to realize the self-replenishment of ILs on the surface. This work provides an opportunity for ionogels to be used as a functional coating with renewable anti-biofouling properties.

In addition to killing bacteria, algae pollution can be resisted by preparing a multi-scale IL polymer brush-modified antifouling surface (He et al., 2021). 1-(1H-Benzotriazolyl) methyl-3norbornene methyl-1H-imidazolium bromide ([BNIm][Br]) was grafted onto the bio-inspired sharklet substrate prepared by 3D printing of acrylic resin (Figure 6E). Compared to the bare sharklet substrate, the removal rate of *Porphyridium* increased from 40.7% to 60.1%. Therefore, ILs can be grafted onto the substrate surface or infused into a porous surface to obtain antibacterial function (Ye et al., 2012), which can be adjusted by the length of the ILs alkyl chain, hydrophobicity, and molecular size.

#### 4.1.2 Anti-liquid fouling

Some organic substances such as protein and grease contained in wastewater will cause deposition on the surface and corrosion of water treatment equipment (Wei et al., 2018), which poses a real threat to the environment (Wei et al., 2022). Many physical and chemical methods (such as distillation and electromagnetic radiation) have been developed for organic pollutants separation in wastewater but have the problems of low efficiency and high energy consumption (Zhang X. et al., 2020). To overcome the defects of the aforementioned methods, the antifouling coating inspired by nature can reduce the deposition of these organic substances and maintain the initial function of the equipment surface.

**FIGURE 7:**
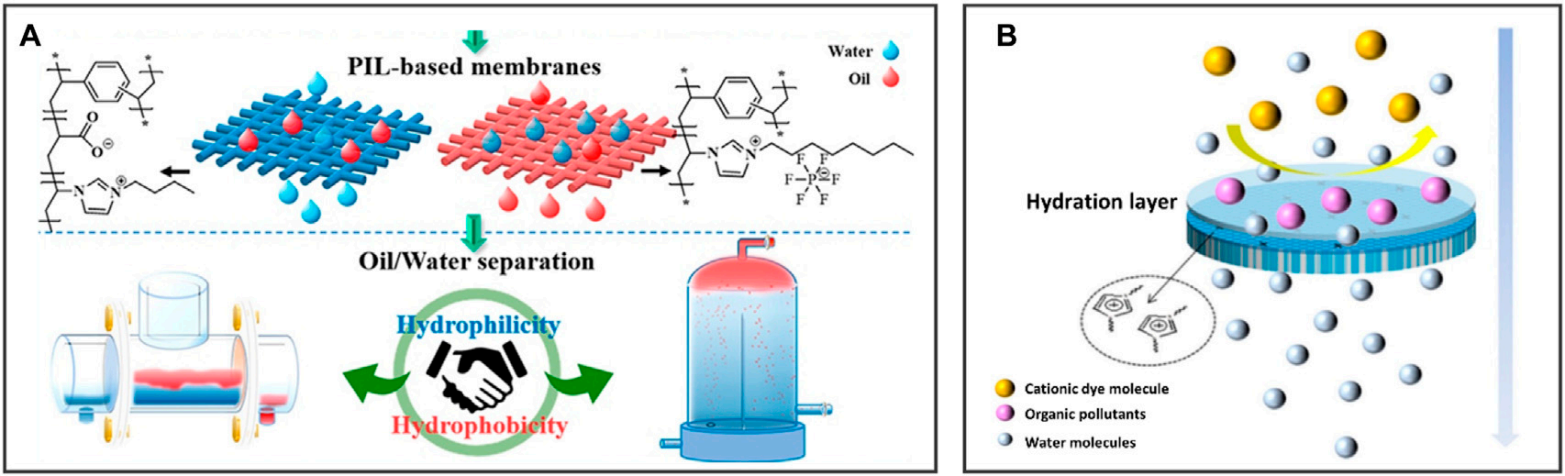
BILIMs for anti-liquid fouling. **(A)** Oil/water separation membrane with adjustable surface wettability realizes continuous oil/water separation without polluted by oil. Reproduced with permission (Deng et al., 2020). Copyright 2020, American Chemical Society. **(B)** ILs modified membrane can separate dyes and purify wastewater containing organic pollutant. Reproduced with permission (Liu et al., 2022). Copyright 2022, Springer Nature.

Deng *et al.* prepared a series of PIL-based oil/water separation membranes with adjustable surface wettability using *N*-vinylimidazolium IL and divinylbenzene (Figure 7A). The hydrophilic poly(1-vinyl-3-butylimidazolium acrylate)-based membrane (PILM-1) transports water and retains oil, while the hydrophobic poly(1-vinyl-3-octylimidazolium hexafluorophosphate)-based membrane (PILM-5) removes oil and retains water. Two types of membranes with opposite wettability show excellent oil/water separation efficiency (over 99%) during the treatment process and realize continuous oil/water separation of 46 L/12 h (Deng et al., 2020). Additionally, Liu et al. (2022) prepared an IL-modified membrane by grafting 1-butyl-3-vinylimidazole bromide IL onto PVDF for improving the wettability and antifouling performance of the membrane (Figure 7B), demonstrating excellent separation performance for cationic dyes in wastewater.

The development of bio-inspired coatings is a promising strategy to solve the increasingly serious problem of organic fouling, which has the advantages of reliable service life, effective cost reduction, and broad application prospects in water treatment equipment.

#### 4.1.3 Anti-solid fouling

Some adhesion of solids (e.g., mineral scale and ice) can pose serious problems to humans in daily production and life. Undesired scale adhesion in pipes will cause fuel waste due to decreased heat transfer efficiency. Furthermore, once ice forms on a surface (e.g., aircraft), it will seriously endanger the normal operation of the equipment and put humans in danger. Therefore, dealing with the solid adhesion problem is of great significance to ensure normal operation. BILIM has achieved success in antifouling and anti-icing applications due to the existence of the liquid barrier layer on the surface.

**FIGURE 8:**
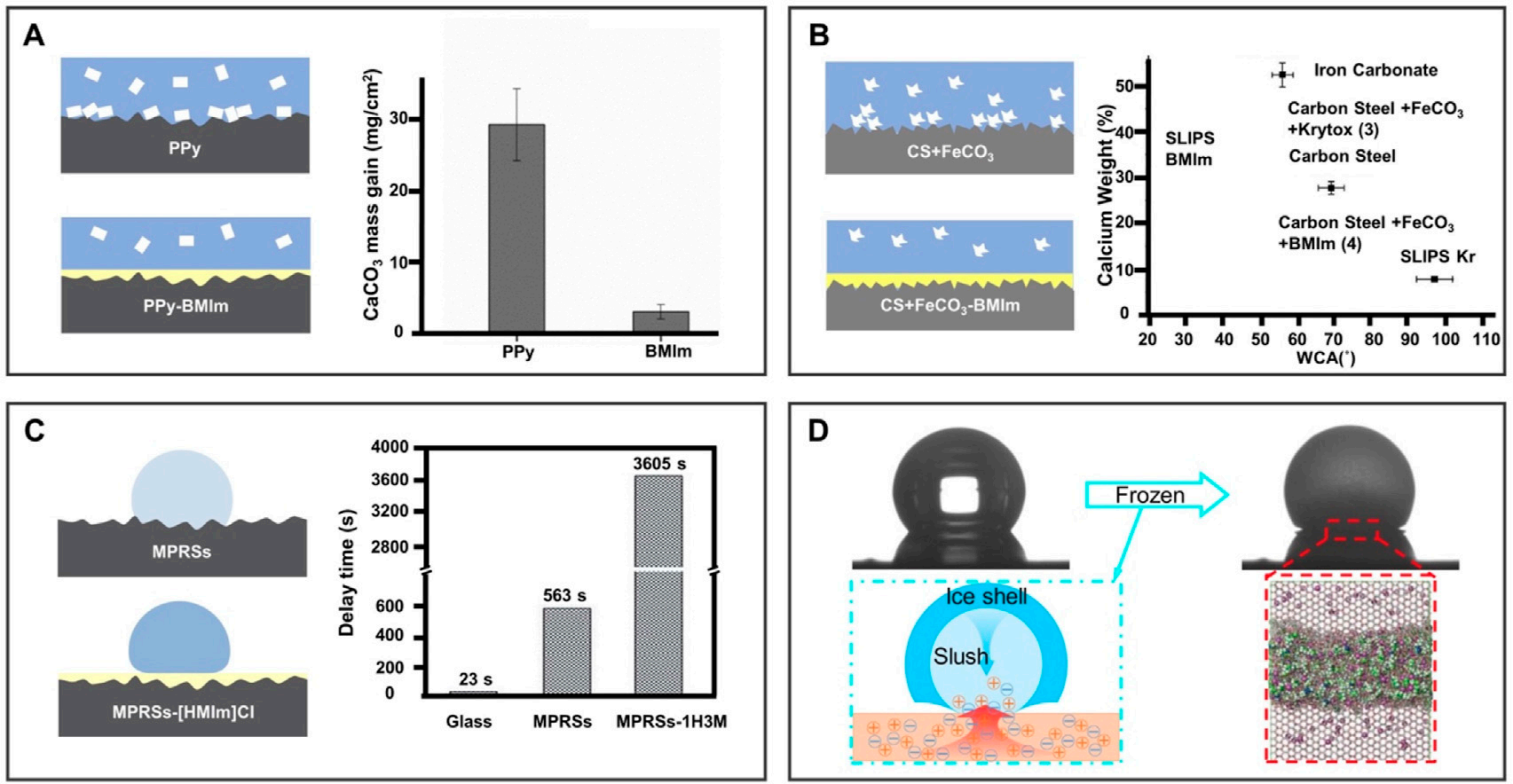
BILIMs for anti-solid fouling. **(A)** Low surface energy lubricant and BMIm were injected into the porous polypyrrole coating to reduce scaling. Reproduced with permission (Charpentier et al., 2015). Copyright 2015, Elsevier Inc. **(B)** BMIm-infused FeCO_3_ layer of carbon steel increases hydrophobicity and reduces scale formation. Reproduced with permission (Saul et al., 2021). Copyright 2021, Informa United Kingdom Limited. **(C)** BMIm[PF_6_] doped polyvinylidene fluoride nanofibers delay the freezing time of water droplets and reduced the crystallization temperature and ice adhesion strength of water droplets. Reproduced with permission (Rao et al., 2019). Copyright 2019, The Royal Society of Chemistry. **(D)** Ionogel surface inhibits ice nucleation, growth, and adhesion. Reproduced with permission (Zhuo et al., 2020). Copyright 2020, American Chemical Society.

Compared to the Fluorinert FC-70-infused surface, the IL-infused surface is more stable on the coating surface because of its moderate surface energy than with fluorinated lubricating oil. Using IL-BMIm as a liquid layer, Charpentier et al. (2015) developed a polypyrrole coating on a stainless-steel substrate using a potentiostatic deposition method in Figure 8A. Due to the slippery and low adhesion properties of the liquid-infused surface, calcium carbonate (CaCO_3_) was significantly reduced by 18 times, offering a novel solution for anti-scaling. In addition, Saul et al. (2021) infused IL-BMIm into the FeCO_3_ layer on X65 carbon steel to prepare a novel kind of BILIM (Figure 8B). There is no need to use a functional layer between the substrate and the lubricant interface and shows enhanced anti-scaling ability against CaCO_3_.

In addition to the adhesion of scales, the adhesion of ice may also cause surface failure or even serious accidents. Therefore, many efforts have been made to reduce the formation and adhesion of ice on the surface. As shown in Figure 8C, the smooth surface obtained by infusing [HMIm]Cl into the polymer has excellent anti-icing performance compared to the bare glass and the polymer surface without an IL infusion. The surface extends the icing time to 3605s and reduces the adhesion of ice (Rao et al., 2019). In addition, Zhuo et al. (2020) developed a novel anti-icing ionogel to inhibit the growth of ice to mitigate the harm caused by accretion ice, as illustrated in Figure 8D. The ionogel demonstrated an outstanding ability to inhibit the growth of ice and prevent the formation of frost in a humidified environment. It is observed that the positive and negative ions of [BMIm]Br separate at the front interface of ice growth, creating an electric field that prevents ice growth due to the formed interface liquid layer with poor adhesion to the substrate.

Although the research on ILs in anti-scaling and anti-icing is small, the existing literature has shown great potential in removing undesired adhesion. It is expected to lead the scientific research wave in anti-scaling and anti-icing by utilizing the lubricating properties and freezing tolerance properties of ILs.

### 4.2 Adhesive materials

There are many interesting adhesion phenomena in nature, such as snails, abalone, mussels, and geckos. Inspired by nature, the interfacial interaction forces formed by the contact between the gecko’s foot and the solid wall provide adhesion for the gecko to walk on a vertical wall, the non-covalent interaction between the adhesive secreted by mussels and the solid substrate is settled on the rock for survival. For BILIMs, their adhesion performance can be adjusted by rationally designing ILs, thus expanding the application range of materials. Due to the high adhesion from the electrostatic effect, IL-based adhesive materials have made innovations in biological sensors, adhesive tape, and wound dressing.

#### 4.2.1 Biological sensor

The rapid rise of the internet has popularized the use of electronic products and improved the life quality of humans. However, traditional electronic devices suffer from limited toughness and poor self-healing properties, while IL-based electronic devices have excellent performances such as flexibility, self-healing property, and reconfigurable property caused by charge effect and high conductivity. Therefore, many research studies have paid attention to building new multi-functional electronic devices based on ILs. The unique mechanical properties of ILs make them promising for various biological sensors.

**FIGURE 9:**
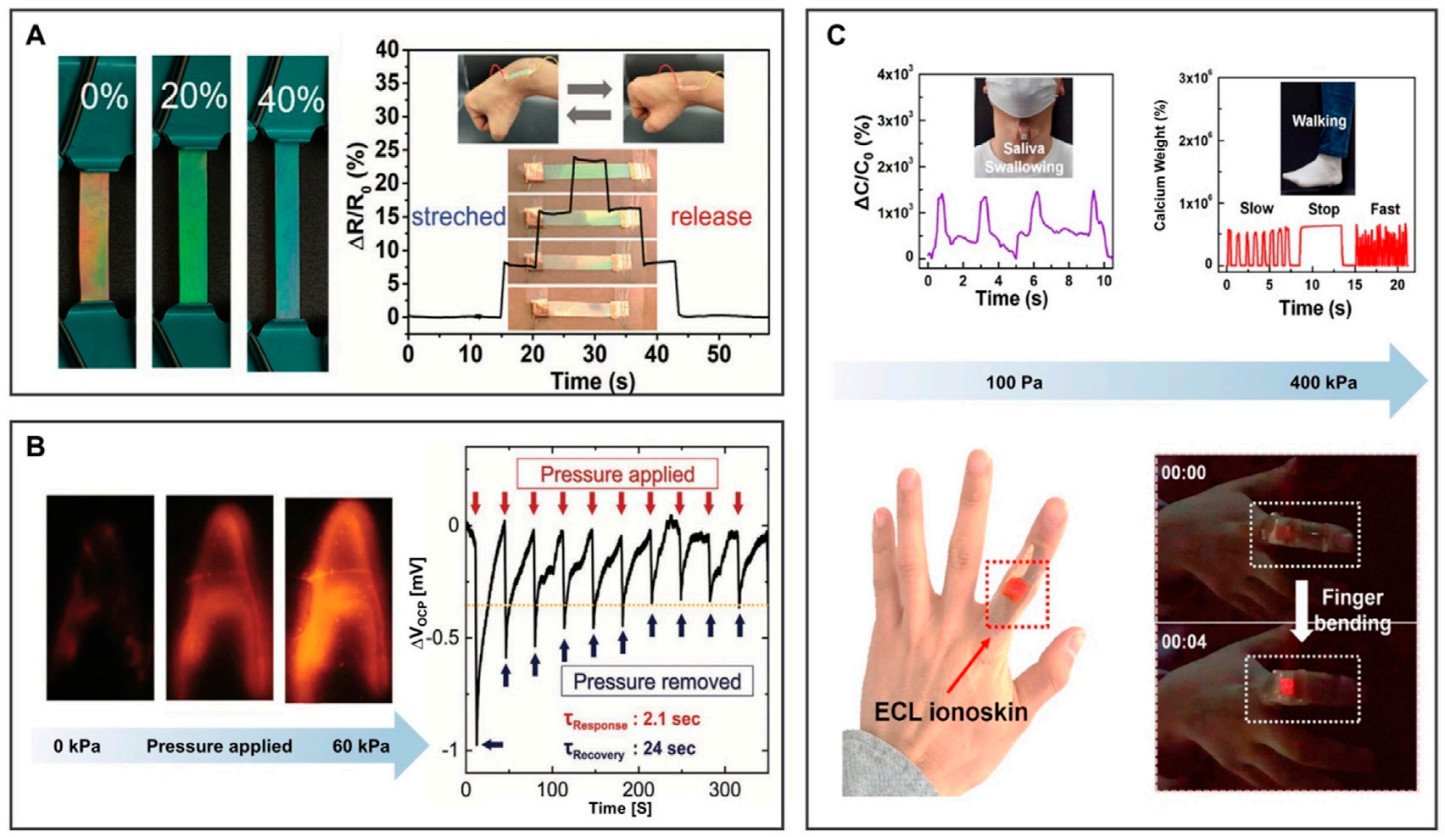
BILIMs for a biological sensor. **(A)** Bionic interactive visible ion skin. Reproduced with permission (Lyu et al., 2021). Copyright 2021, Wiley-VCH GmbH. **(B)** Electrochemiluminescence skin with the piezoelectric ion effect. Reproduced with permission (Lee et al., 2021). Copyright 2021, Wiley-VCH GmbH. **(C)** Photoelectric dual output high-performance wearable ion skin. Reproduced with permission (Kwon et al., 2021). Copyright 2021, American Chemical Society.

To obtain photonic ionogels (PIGs) with good stability and synergistic sensitivity, Lyu et al. (2021) developed an ion skin by locking 1-ethyl-3-methylimidazolium bis-(trifluoromethylsulfonyl)imide ([EMIm][TFSI]) into poly(ethylene glycol) phenyl ether acrylate (PEGPEA) polymer elastomer as shown in Figure 9A. Under a slight tensile strain, the PIGs immediately changed from orange to green. When the strain reached 40%, the PIGs changed to blue, showing excellent mechanochromic performance. They can be used for ion skin sensors and photoelectric interactive devices.

The deformable electronic skin device was designed to convert local stress into a spatially resolved optical signal (Lee et al., 2021). The electrochemical performance of the composite material is controlled by the electrochemiluminescence (ECL)-active material (ECL: Ru(bpy)_3_)[PF_6_]_2_, IL: [EMIm][TFSI]), and the mechanical performance is controlled by polyurethane matrix (Figure 9B). When pressure is applied to the film from the top, cations leave and anions are retained due to the difference in the mobility rate of anions and cations, thereby appearing negative charge layer and positive charge layer on the film. When the pressure was removed, the electrical potential will slowly return to its original value until a uniform ion distribution is obtained. For the aforementioned reasons, the ECL skin device platform can convert mechanical stimuli into visual readings, laying the foundation for the design of tactile sensors for human–machine interaction with electronic skin. In addition, the structural changes of porous ionogels can also be used to visualize electronic signals. As described in Figure 9C, Kwon et al. (2021) prepared porous ionogel by *in situ* crosslinking polymerization of PEA-g-PS-g-PDVB and [EMIm][TFSI]. After applying pressure, the elastic porous ionogel collapsed and the pores closed. Therefore, the contact area between the gel and the electrode increases, resulting in a higher ECL capacitance. The applied pressure can be directly displayed by the brightness of the emitted light, and it is expected to become an important part of high-performance, functional ion electronics. Additionally, molding soft BILIMs into specific structures with strong adhesion and high conductivity by utilizing 3D printing has prospects for intelligent devices such as biosensors, wearable electronic devices, and smart robotics (Chen et al., 2022).

Electronic devices based on ILs have made innovations in the development and application of flexible wearable devices (Wang et al., 2022). IL-based electronic devices are mainly in the form of ionogel, which confines the ILs in an elastic polymer matrix. ILs have excellent properties such as conductivity and electrochemical stability, thereby improving the electrochemical performance of electronic devices. In addition, conductive additives such as carbon-based materials and metal nanoparticles were introduced into the polymer network to improve high conductivity and mechanical properties. However, most of the research is still in the theoretical research or basic research stage and generally has problems with stability and durability. The future research direction is to continue to optimize the performance of biological devices to obtain high-performance devices.

#### 4.2.2 Adhesive tape

Underwater adhesion generally has problems such as poor adhesion and instability, which seriously affect the efficacy and service life of adhesive materials in practical applications. IL-based adhesive tape has broad application prospects due to its underwater stability and adhesion, the adhesive strength of which can be adjusted by changing the types of ILs. In addition, physical interaction can be introduced into adhesive tape based on ionogel to further enhance the adhesion strength.

**FIGURE 10:**
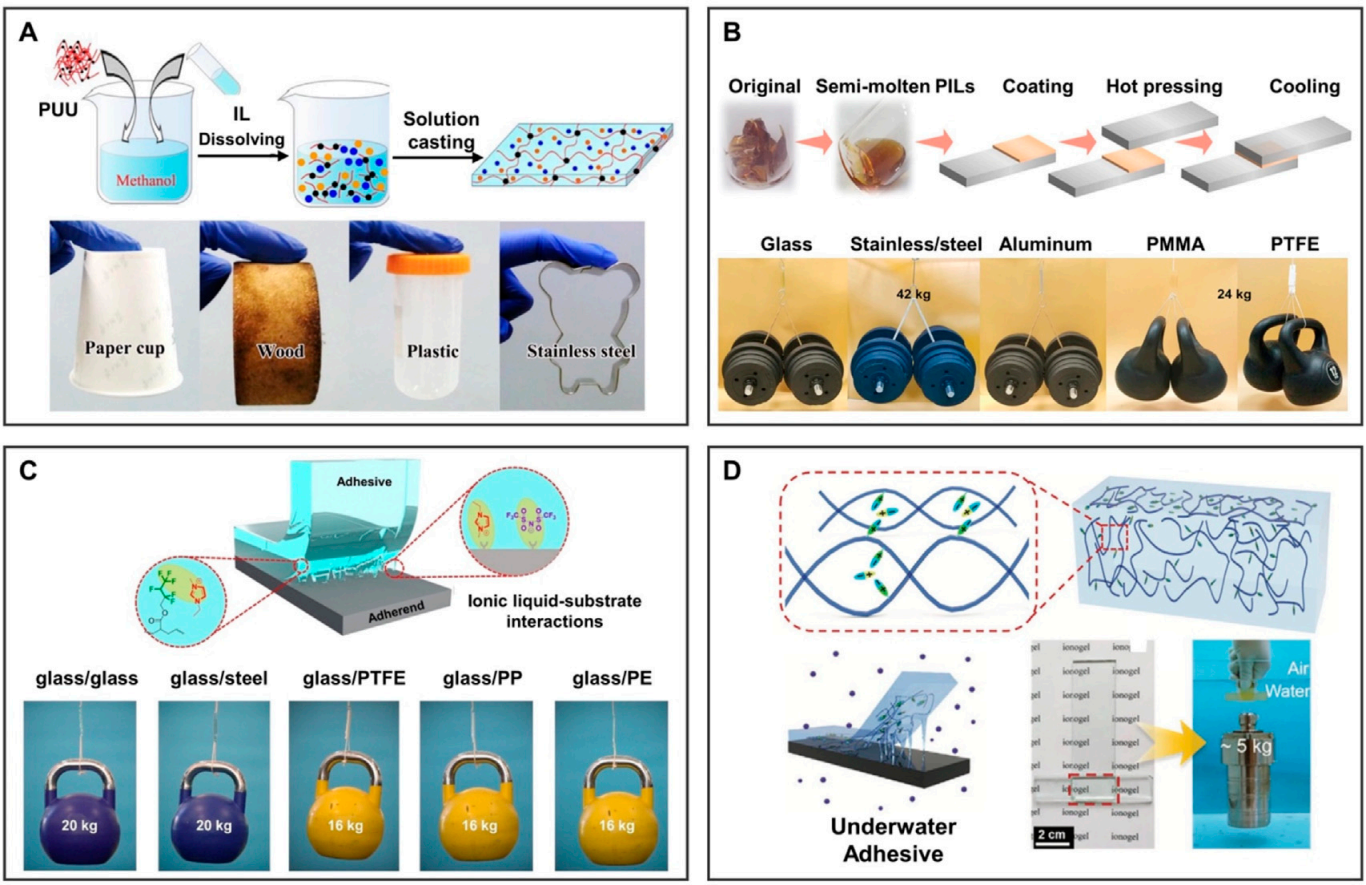
BILIMs for adhesive tape. **(A)** PUU polymer hydrogen bonding and amphiphilicity enable ionogels to adhere to any substrate, thus realizing the university of adherence to substrates. Reproduced with permission (Chen and Guo, 2021). Copyright 2021, American Chemical Society. **(B)** Flexible alkoxy chains are introduced into the cationic backbone of PILs, enabling strong hydrogen bonding and electrostatic interactions while contributing to high cohesive energy and interfacial adhesive energy to obtain high-efficiency adhesive. Reproduced with permission (Zhang et al., 2021). Copyright 2021, Wiley-VCH GmbH. **(C)** Ion-dipole interactions between P(HFBA-*co*-MMA) and hydrophobic ILs for higher adhesion strength and reversible adhesion. Reproduced with permission (Huang et al., 2021). Copyright 2021, American Chemical Society. **(D)** Ionogel soaked in the salt solution can discharge the salt ions on the surface of the substrate to adhere to the substrate, and the adhered substrate can lift a heavy object up to 5 kg, realizing reversible and strong adhesion. Reproduced with permission (Yu and Wu, 2021). Copyright 2021, Wiley-VCH GmbH.

As a common physical interaction, hydrogen bonds can be used to enhance the adhesion of the tape. As shown in Figure 10A, Guo et al.2020 developed supramolecular hydrogel containing poly(urethane-urea) (PUU) and ILs (Chen 2021 Guo, 2021). The hydrogen bond endows the ionogel with excellent adhesion, which can be adhered to any substrate. It can be attached to a human’s skin as a sensor to monitor various movements. Additionally, an efficient adhesive tape was prepared by simply introducing flexible alkoxy into the cationic skeleton of PILs containing TFSI^−^ anion (Figure 10B). The strong hydrogen bond and electrostatic interaction between flexible alkoxy chains and PILs both contribute to high cohesive energy and interfacial adhesion energy (Zhang et al., 2021). Alkoxy PILs can adhere to various substrates such as glass, ceramic, and stainless steel.

In addition to the hydrogen bond, the effect of a charge also enhances adhesion. The transparent ionogel adhesive tape was composed of hydrophobic ILs and poly(hexafluorobutyl acrylate-*co*-methyl methacrylate) (Huang et al., 2021). The ionogel adhesive tape achieved high adhesive strength through an ion-dipole interaction, which can be reversibly adhered to various substrates, such as glass, steel, and PTFE, and the adhesive strength of ionogel was higher than that of most commercial adhesive tapes (Figure 10C). Cations of the ionogel can repel salt ions from the substrate surface, and quickly and firmly adhere to various substrates in salt solution (Yu and Wu, 2021) (Figure 10D). The ionogel possesses the performance of high ionic conductivity, underwater adhesion, self-healing, and stretchability, which can lift an object up to 5 kg underwater and repeatedly adhere to the substrate for sensors.

IL-based adhesive tapes take full advantage of the characteristics of ILs and polymer networks to obtain high adhesive strength through physical interaction including hydrogen bonds and charge interaction. The adhesive strength, self-healing ability, stretchability, optical transparency, electrical conductivity, and other properties of IL-based adhesive tape can be controlled by reasonably designing the structure of ILs.

#### 4.2.3 Wound dressing

**FIGURE 11:**
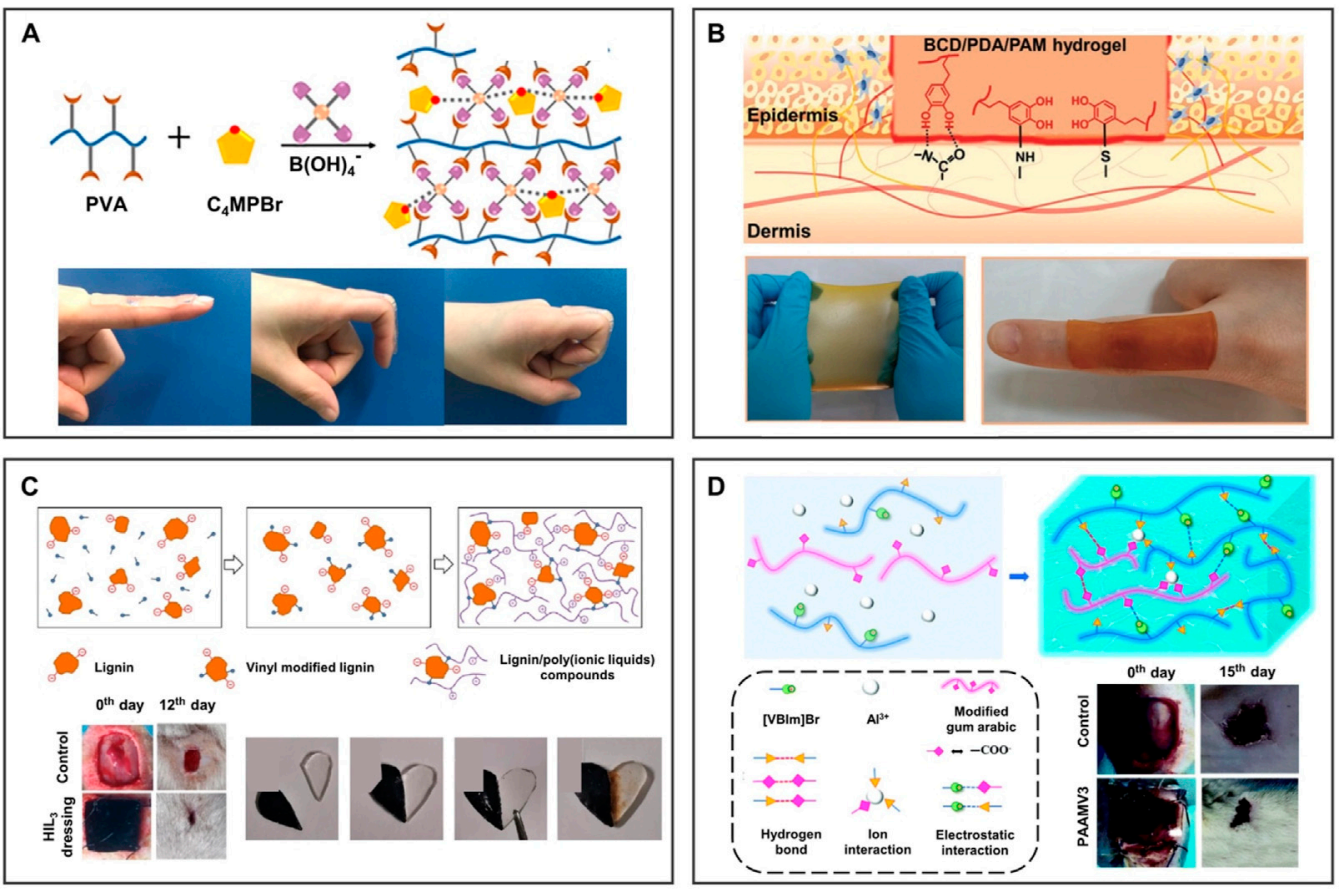
BILIMs for wound dressing. **(A)** Incorporation of pyrrolidine to provide ionogel adhesion for application in wound dressings. Reproduced with permission (Yu et al., 2020). Copyright 2020, Elsevier B.V. **(B)** After the introduction of PDA containing abundant catechol groups, ionogel can be attached to the areas where the body moves greatly and realize the treatment of special regional wounds. Reproduced with permission (Yang et al., 2021). Copyright 2021, Wiley-VCH GmbH. **(C)** Lignin with a three-dimensional network structure and antioxidant activity was introduced into ionogel, which showed good antibacterial activity and promoted wound healing. Reproduced with permission (Zhang X. et al., 2020). Copyright 2020, Elsevier B.V. **(D)** After combining ILs with hydrogel dressing containing migratable ions, dressing promotes the migration of ions, thus promoting self-healing performance. Reproduced with permission (Li et al., 2021). Copyright 2021, The Royal Society of Chemistry.

Many efforts have been made to apply adhesive materials to wound dressings; however, little attention has been paid to combining high adhesion with antibacterial/antiinflammatory functions in existing solutions. The incorporation of ILs brings high adhesion to antibacterial wound dressings, which makes up for the aforementioned problem. As shown in Figure 11A, Yu et al. (2020) designed an antibacterial hydrogel containing pyrrole ILs, which showed effective antibacterial activity against Gram-negative and Gram-positive bacteria. The antibacterial and adhesive properties of ionogel give it potential in wound dressing applications. Inspired by the bio-adhesion of mussel dopamine, Yang et al. (2021) fabricated an adhesive hydrogel dressing containing PDA. As shown in Figure 11B, PDA components are rich in catechol groups, so they can adhere to various surfaces, especially in special areas requiring large movements, which can be used as a wound dressing. Inspired by trees, Zhang Y. et al. (2020) synthesized a novel type of lignin/PILs composite hydrogel dressing. As shown in Figure 11C, self-healing hydrogel obtained by the supramolecular interaction between lignin and PILs can promote wound healing in rats. The introduction of lignin with a three-dimensional network structure can significantly improve the mechanical property and antioxidant activity of hydrogel dressing. The IL-based hydrogel dressing with excellent antibacterial performance can promote skin wound healing wonderfully. As depicted in Figure 11D, (Li et al. (2021) grafted positively charged IL 1-vinyl-3-butylimidazolium ([VBIm]Br) and Al^3+^ onto the main chain of hydrogel through covalent interaction. On the one hand, ILs can promote the hydrophobic interaction between hydrophobic segments of hydrogel, which can show the good mechanical properties of hydrogel after absorbing water. On the other hand, charged ILs can promote the migration of Al^3+^, improving the self-healing performance of hydrogel and accelerating the self-healing of rat wounds. The synergistic effect of strong adhesion and antibacterial properties of the IL-based wound dressing will play a huge role in wound healing.

Considering the biosafety of BILIMs in bio-adhesion and wound dressing, numerous studies have shown that choline, some ammonium ions, and glycine betaine can be utilized as cations to obtain biological ILs with biodegradable and low toxicity (Gomes et al., 2019). IL-based wound dressing possesses a good bactericidal effect on common bacilli, cocci, and fungi, which has good biocompatibility without destroying the normal cell structure. In addition, the introduction of charged ILs endows IL-based wound dressing with good mechanical properties and adhesion, which is conducive to promoting the self-healing of the wound. Therefore, BILIM provides a broad application prospect in wound dressing.

## 5 Summary and outlook

**FIGURE 12:**
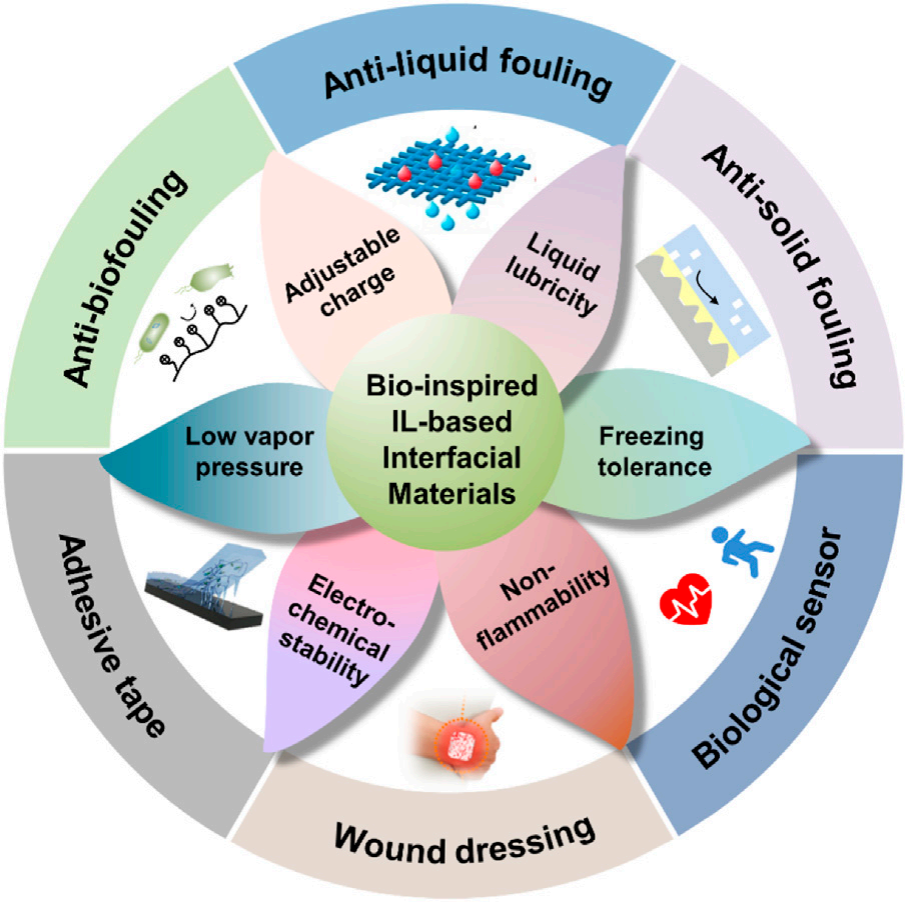
Unique properties and applications of BILIMs. BILIM inherits the advantages of ILs, such as charge adjustability and electrochemical stability, and shows promising potential applications in anti-adhesion (anti-biofouling, anti-liquid fouling, and anti-solid fouling) and adhesion (biological sensor, adhesive tape, and wound dressing). Reproduced with permission (Zhang et al., 2019). Copyright 2019, American Chemical Society. Reproduced with permission (Cho et al., 2020). Copyright 2020, WILEY-VCH Verlag GmbH & Co. KGaA, Weinheim. Reproduced with permission (Wang et al., 2020). Copyright 2020, American Chemical Society. Reproduced with permission (Yao et al., 2022). Copyright 2022, Wiley-VCH GmbH (Deng et al., 2020). Copyright 2020, American Chemical Society.

Due to the negligible vapor pressure, adjustable charge, liquid lubricity, freezing tolerance, non-flammability, and electro-chemical stability of ILs (Figure 12), BILIMs have attracted the interest of scientific researchers, making them considerably important in antifouling, anti-liquid fouling, anti-solid fouling, adhesive tape, wound dressing, and biological sensors. Although great achievements have been made in various applications of ILs, there are still some challenges to be solved in BILIMs.

Although BILIMs have been used for anti-microbial purposes, the mechanism is still unclear: the increase in the alkyl chain length of ILs does not increase the antibacterial ability for all bacterial species, and the effect of structural changes on their antimicrobial capacity has not been widely discussed. The currently accepted mechanism is the interaction between the negatively charged bacterial cell membrane and the positive charges of the ILs disturbing the cell membrane, the hydrophobic segment of the ILs is inserted into the cell membrane, resulting in the death of bacteria. However, this theory has not yet been confirmed, and the antibacterial mechanism of ILs should be clarified in the future. Of course, with IL as an ideal lubrication layer, the loss of the IL layer on the porous surface is an inevitable problem when it is in a complex fluid environment. In future development, improving the stability and durability of the IL-infused coating is the primary problem to be solved and may be considered from the aspects of intelligent control of the state of the IL layer on the surface and intelligent release.

The existing IL-based electronic devices may have undesired chemical reactions under high pressure, which may cause a decrease in conductivity and short circuits, thus leading to the failure of these electronic devices. Encapsulating ILs can alleviate this failure mechanism to some extent, yet brings problems of compatibility with the package interface, such as poor adhesion, poor toughness, and difficulty in bonding. Therefore, it is necessary to further explore the internal composition and crosslinking forms of ILs materials to improve the electrochemical performance, durability, stability, and compatibility of biological sensors.

Due to the diversity and the adjustment of anions and cations, IL-based stimuli-responsive materials can achieve controllable physical and chemical properties as well as functions by reasonably designing ionic species anion, cation, hydrophilicity, and hydrophobicity of ILs. Although many efforts have been made to develop various IL-based smart devices, it is necessary to systematically study the mechanism between the properties of ILs and the stimuli-responsive performance of ILs. From the perspective of practical applications, there are still many challenges and opportunities.

## References

[B1] Agarwal H. Nyffeler K. E. Blackwell H. E. Lynn D. M. (2021). Fabrication of slippery liquid-infused coatings in flexible narrow-bore tubing. ACS Appl. Mat. Interfaces 13, 55621–55632. 10.1021/acsami.1c14662 PMC8840327 34775755

[B2] Bechert D. W. Bruse M. Hage W. VanderHoeven J. G. T. Hoppe G. (1997). Experiments on drag-reducing surfaces and their optimization with an adjustable geometry. J. Fluid Mech. 338, 59–87. 10.1017/s0022112096004673

[B3] Branco L. C. Crespo J. G. Afonso C. A. M. (2002). Highly selective transport of organic compounds by using supported liquid membranes based on ionic liquids. Angew. Chem. Int. Ed. 41, 2771–2773. 10.1002/1521-3773(20020802)41:15<2771:aid-anie2771>3.0.co;2-u 12203480

[B4] Buddingh J. V. Hozumi A. Liu G. (2021). Liquid and liquid-like surfaces/coatings that readily slide fluids. Prog. Polym. Sci. 123, 101468–101497. 10.1016/j.progpolymsci.2021.101468

[B5] Cao Y. Morrissey T. G. Acome E. Allec S. I. Wong B. M. Keplinger C. (2017). A transparent, self-healing, highly stretchable ionic conductor. Adv. Mat. 29, 1605099–1605107. 10.1002/adma.201605099 28009480

[B6] Cao Z. Liu H. Jiang L. (2020). Transparent, mechanically robust, and ultrastable ionogels enabled by hydrogen bonding between elastomers and ionic liquids. Mat. Horiz. 7, 912–918. 10.1039/c9mh01699f

[B7] Carlin R. Fuller J. (1997). Ionic liquid-polymer gel catalytic membrane. Chem. Commun. 15, 1345–1346. 10.1039/a702195j

[B8] Charpentier T. V. Neville A. Baudin S. Smith M. J. Euvrard M. Bell A. (2015). Liquid infused porous surfaces for mineral fouling mitigation. J. Colloid Interface Sci. 444, 81–86. 10.1016/j.jcis.2014.12.043 25585291

[B9] Chen H. Ge P. Yan Z. Chen M. Dai X. Zhuo H. (2022). 3D printable, biomimetic adhesive, and self-healing acrylic elastomers for customized attachable strain sensor. Chem. Eng. J. 430, 133111–133120. 10.1016/j.cej.2021.133111

[B10] Chen H. Zhang P. Zhang L. Liu H. Jiang Y. Zhang D. (2016). Continuous directional water transport on the peristome surface of Nepenthes alata. Nature 532, 85–89. 10.1038/nature17189 27078568

[B11] Chen L. Guo M. (2021). Highly transparent, stretchable, and conductive supramolecular ionogels integrated with three-dimensional printable, adhesive, healable, and recyclable character. ACS Appl. Mat. Interfaces 13, 25365–25373. 10.1021/acsami.1c04255 34003634

[B12] Cheng Y. Y. Du C. H. Wu C. J. Sun K. X. Chi N. P. (2018). Improving the hydrophilic and antifouling properties of poly(vinyl chloride) membranes by atom transfer radical polymerization grafting of poly(ionic liquid) brushes. Polym. Adv. Technol. 29, 623–631. 10.1002/pat.4172

[B13] Cho K. Kim H. Jang S. Kyung H. Kang M. Lee K. (2020). Optimizing electrochemically active surfaces of carbonaceous electrodes for ionogel based supercapacitors. Adv. Funct. Mat. 30, 2002053. 10.1002/adfm.202002053

[B14] Cui Y. Gong H. Wang Y. Li D. Bai H. (2018). A thermally insulating textile inspired by polar bear hair. Adv. Mat. 30, e1706807. 10.1002/adma.201706807 29443435

[B15] Demirci S. Kinali-Demirci S. VanVeller B. (2020). Surface-grafted polymeric ionic liquids with tunable morphology *via* in/*ex situ* cross-linking methods. ACS Macro Lett. 9, 1806–1811. 10.1021/acsmacrolett.0c00632 35653685

[B16] Deng X. Zhang J. Zhang L. Cheng G. Chen B. Zhang Y. (2020). Poly(ionic liquid)-coated meshes with opposite wettability for continuous oil/water separation. Ind. Eng. Chem. Res. 59, 6672–6680. 10.1021/acs.iecr.0c00554

[B17] Ding Y. Zhang J. Chang L. Zhang X. Liu H. Jiang L. (2017). Preparation of high-performance ionogels with excellent transparency, good mechanical strength, and high conductivity. Adv. Mat. 29, 1704253–1704259. 10.1002/adma.201704253 29083496

[B18] Dou H. Xu M. Wang B. Zhang Z. Luo D. Shi B. (2021). Analogous mixed matrix membranes with self-assembled interface pathways. Angew. Chem. Int. Ed. 60, 5864–5870. 10.1002/anie.202014893 33170995

[B19] Feng L. Li S. H. Li Y. S. Li H. J. Zhang L. J. Zhai J. (2002). Super-hydrophobic surfaces: From natural to artificial. Adv. Mat. 14, 1857–1860. 10.1002/adma.200290020

[B20] Fuller J. Breda A. C. Carlin R. T. (1997). Ionic liquid-polymer gel electrolytes. J. Electrochem. Soc. 144, L67–L70. 10.1149/1.1837555

[B21] Gomes J. M. Silva S. S. Reis R. L. (2019). Biocompatible ionic liquids: Fundamental behaviours and applications. Chem. Soc. Rev. 48, 4317–4335. 10.1039/c9cs00016j 31225558

[B22] Guo W. Mahurin S. M. Unocic R. R. Luo H. Dai S. (2020). Broadening the gas separation utility of monolayer nanoporous graphene membranes by an ionic liquid gating. Nano Lett. 20, 7995–8000. 10.1021/acs.nanolett.0c02860 33064492

[B23] He B. Du Y. Wang B. Wang X. Ye Q. Liu S. (2021). Grafting embedded poly(ionic liquid) brushes on biomimetic sharklet resin surface for anti-biofouling applications. Prog. Org. Coat. 157, 106298–106305. 10.1016/j.porgcoat.2021.106298

[B24] Hirota Y. Maeda Y. Yamamoto Y. Miyamoto M. Nishiyama N. (2017). Organosilica membrane with ionic liquid properties for separation of toluene/H_2_ mixture. Materials 10, 901–907. 10.3390/ma10080901 28771202 PMC5578267

[B25] Howell C. Grinthal A. Sunny S. Aizenberg M. Aizenberg J. (2018). Designing liquid-infused surfaces for medical applications: A review. Adv. Mat. 30, e1802724. 10.1002/adma.201802724 30151909

[B26] Huang S. Wan Y. Ming X. Zhou J. Zhou M. Chen H. (2021). Adhering low surface energy materials without surface pretreatment via ion-dipole interactions. ACS Appl. Mat. Inter. 13, 41112–41119. 10.1021/acsami.1c11822 34406738

[B27] Jin H. Tian L. Bing W. Zhao J. Ren L. (2022). Bioinspired marine antifouling coatings: Status, prospects, and future. Prog. Mat. Sci. 124, 100889–100941. 10.1016/j.pmatsci.2021.100889

[B28] Ju J. Bai H. Zheng Y. Zhao T. Fang R. Jiang L. (2012). A multi-structural and multi-functional integrated fog collection system in cactus. Nat. Commun. 3, 1247–1252. 10.1038/ncomms2253 23212376 PMC3535335

[B29] Kwon J. H. Kim Y. M. Moon H. C. (2021). Porous ion gel: A versatile ionotronic sensory platform for high-performance, wearable ionoskins with electrical and optical dual output. ACS Nano 15, 15132–15141. 10.1021/acsnano.1c05570 34427425

[B30] Laloyaux X. Fautre E. Blin T. Purohit V. Leprince J. Jouenne T. (2010). Temperature-responsive polymer brushes switching from bactericidal to cell-repellent. Adv. Mat. 22, 5024–5028. 10.1002/adma.201002538 20734384

[B31] Lan J. Li Y. Yan B. Yin C. Ran R. Shi L. Y. (2020). Transparent stretchable dual-network ionogel with temperature tolerance for high-performance flexible strain sensors. ACS Appl. Mat. Interfaces 12, 37597–37606. 10.1021/acsami.0c10495 32700894

[B32] Lee J. I. Choi H. Kong S. H. Park S. Park D. Kim J. S. (2021). Visco-poroelastic electrochemiluminescence skin with piezo-ionic effect. Adv. Mat. 33, e2100321. 10.1002/adma.202100321 34060148

[B33] Li D. Fei X. Wang K. Xu L. Wang Y. Tian J. (2021). A novel self-healing triple physical cross-linked hydrogel for antibacterial dressing. J. Mat. Chem. B 9, 6844–6855. 10.1039/d1tb01257f 34612333

[B34] Li H. Yan M. Zhao W. (2022). Designing a MOF-based slippery lubricant-infused porous surface with dual functional anti-fouling strategy. J. Colloid Interface Sci. 607, 1424–1435. 10.1016/j.jcis.2021.09.052 34583045

[B35] Li J. L. Zhang Y. Zhang S. Liu M. Li X. Cai T. (2019). Hyperbranched poly(ionic liquid) functionalized poly(ether sulfone) membranes as healable antifouling coatings for osmotic power generation. J. Mat. Chem. A 7, 8167–8176. 10.1039/c8ta10484k

[B36] Liang C. D. Yuan C. Y. Warmack R. J. Barnes C. E. Dai S. (2002). Ionic liquids: A new class of sensing materials for detection of organic vapors based on the use of a quartz crystal microbalance. Anal. Chem. 74, 2172–2176. 10.1021/ac011007h 12033323

[B37] Liu H. Xie J. Zhao J. Xue P. Lv X. Sun S. (2022). Hydrophilic ionic-liquid grafted poly(vinylidene fluoride) membranes with excellent cationic dye and oil-water emulsion removal performance. J. Mat. Sci. 57, 4876–4894. 10.1007/s10853-022-06911-8

[B38] Liu L. Xiong S. Zeng L. Cai C. Li F. Tan Z. (2021). Two birds with one stone: Porous poly(ionic liquids) membrane with high efficiency for the separation of amino acids mixture and its antibacterial properties. J. Colloid Interface Sci. 584, 866–874. 10.1016/j.jcis.2020.10.018 33097225

[B39] Liu Y. Wang W. Gu K. Yao J. Shao Z. Chen X. (2021). Poly(vinyl alcohol) hydrogels with integrated toughness, conductivity, and freezing tolerance based on ionic liquid/water binary solvent systems. ACS Appl. Mat. Interfaces 13, 29008–29020. 10.1021/acsami.1c09006 34121382

[B40] Lu J. Yan F. Texter J. (2009). Advanced applications of ionic liquids in polymer science. Prog. Polym. Sci. 34, 431–448. 10.1016/j.progpolymsci.2008.12.001

[B41] Lyu Q. Wang S. Peng B. Chen X. Du S. Li M. (2021). Bioinspired photonic ionogels as interactively visual ionic skin with optical and electrical synergy. Small 17, e2103271. 10.1002/smll.202103271 34510737

[B42] Mantravadi R. Chinnam P. R. Dikin D. A. Wunder S. L. (2016). High conductivity, high strength solid electrolytes formed by *in situ* encapsulation of ionic liquids in nanofibrillar methyl cellulose networks. ACS Appl. Mat. Interfaces 8, 13426–13436. 10.1021/acsami.6b02903 27153318

[B43] Men Y. Drechsler M. Yuan J. (2013). Double-stimuli-responsive spherical polymer brushes with a poly(ionic liquid) core and a thermoresponsive shell. Macromol. Rapid Commun. 34, 1721–1727. 10.1002/marc.201300628 24186465

[B44] Meng J. Wang S. (2019). Advanced antiscaling interfacial materials toward highly efficient heat energy transfer. Adv. Funct. Mat. 30, 1904796–1904813. 10.1002/adfm.201904796

[B45] Muldoon M. Gordon C. (2004). Synthesis of gel-type polymer beads from ionic liquid monomers. Sci. Pol. Chem. 42, 3865–3869. 10.1002/pola.20299

[B46] Njoroge I. Matson M. W. Jennings G. K. (2017). Dynamic anion-adaptive poly(ionic liquid) films via surface-initiated ring-opening metathesis polymerization. J. Phys. Chem. C 121, 20323–20334. 10.1021/acs.jpcc.7b05834

[B47] Noble R. D. Gin D. L. Gin D. L. (2011). Perspective on ionic liquids and ionic liquid membranes. J. Membr. Sci. 369, 1–4. 10.1016/j.memsci.2010.11.075

[B48] Ohno H. (2007). Design of ion conductive polymers based on ionic liquids. Macromol. Symp. 249, 551–556. 10.1002/masy.200750435

[B49] Peppou-Chapman S. Hong J. K. Waterhouse A. Neto C. (2020). Life and death of liquid-infused surfaces: A review on the choice, analysis and fate of the infused liquid layer. Chem. Soc. Rev. 49, 3688–3715. 10.1039/d0cs00036a 32396597

[B50] Qin J. Guo J. Xu Q. Zheng Z. Mao H. Yan F. (2017). Synthesis of pyrrolidinium-type poly(ionic liquid) membranes for antibacterial applications. ACS Appl. Mat. Interfaces 9, 10504–10511. 10.1021/acsami.7b00387 28272866

[B51] Rao Q. Li A. Zhang J. Jiang J. Zhang Q. Zhan X. (2019). Multi-functional fluorinated ionic liquid infused slippery surfaces with dual-responsive wettability switching and self-repairing. J. Mat. Chem. A 7, 2172–2183. 10.1039/c8ta08956f

[B52] Ren Y. Guo J. Liu Z. Sun Z. Wu Y. Liu L. (2019). Ionic liquid–based click-ionogels. Sci. Adv. 5, eaax0648. 10.1126/sciadv.aax0648 31467977 PMC6707778

[B54] Rynkowska E. Dzieszkowski K. Lancien A. Fatyeyeva K. Szymczyk A. Kujawa J. (2017). Physicochemical properties and pervaporation performance of dense membranes based on cellulose acetate propionate (CAP) and containing polymerizable ionic liquid (PIL). J. Membr. Sci. 544, 243–251. 10.1016/j.memsci.2017.09.031

[B55] Salbaum T. Galvan Y. Haumann M. Wasserscheid P. Zarraga R. Vogel N. (2021). Enduring liquid repellency through slippery ionic liquid-infused organogels. J. Mat. Chem. A 9, 2357–2366. 10.1039/d0ta10237g

[B56] Sasikumar B. Arthanareeswaran G. Ismail A. F. (2018). Recent progress in ionic liquid membranes for gas separation. J. Mol. Liq. 266, 330–341. 10.1016/j.molliq.2018.06.081

[B57] Saul A. Barker R. Baraka-Lokmane S. Le Beulze A. Charpentier T. Tangparitkul S. (2021). Corrosion derived lubricant infused surfaces on X65 carbon steel for improved inorganic scaling performance. J. Adhes. Sci. Technol. 36, 632–722. 10.1080/01694243.2021.1932315

[B58] Singh G. Singh G. Damarla K. Sharma P. K. Kumar A. Kang T. S. (2017). Gelatin-based highly stretchable, self-healing, conducting, multiadhesive, and antimicrobial ionogels embedded with Ag_2_O nanoparticles. ACS Sustain. Chem. Eng. 5, 6568–6577. 10.1021/acssuschemeng.7b00719

[B59] Smith J. D. Dhiman R. Anand S. Reza-Garduno E. Cohen R. E. McKinley G. H. (2013). Droplet mobility on lubricant-impregnated surfaces. Soft Matter 9, 1772–1780. 10.1039/c2sm27032c

[B60] Sprugis E. Vaivars G. Merijs Meri R. (2019). A study of mechanical properties of polymer composite membranes with various ionic liquids at elevated temperatures. Mat. Sci. 25, 66–70. 10.5755/j01.ms.25.1.18933

[B61] Sun N. Sun P. Wu A. Qiao X. Lu F. Zheng L. (2018). Facile fabrication of thermo/redox responsive hydrogels based on a dual crosslinked matrix for a smart on-off switch. Soft Matter 14, 4327–4334. 10.1039/c8sm00504d 29761197

[B62] Wang K. Wang J. Li L. Xu L. Feng N. Wang Y. (2020). Novel nonreleasing antibacterial hydrogel dressing by a one-pot method. ACS Biomater. Sci. Eng. 6, 1259–1268. 10.1021/acsbiomaterials.9b01812 33464855

[B63] Wang X. Gu C. Wang L. Zhang J. Tu J. (2018). Ionic liquids-infused slippery surfaces for condensation and hot water repellency. Chem. Eng. J. 343, 561–571. 10.1016/j.cej.2018.03.045

[B64] Wang Z. Cui H. Liu M. Grage S. L. Hoffmann M. Sedghamiz E. (2022). Tough, transparent, 3D-printable, and self-healing poly(ethylene glycol)-gel (PEGgel). Adv. Mat. 34, e2107791–e2107804. 10.1002/adma.202107791 34854140

[B65] Wang Z. Liu Y. Guo P. Heng L. Jiang L. (2018). Photoelectric synergetic responsive slippery surfaces based on tailored anisotropic films generated by interfacial directional freezing. Adv. Funct. Mat. 28, 1801310–1801319. 10.1002/adfm.201801310

[B66] Washiro S. Yoshizawa M. Nakajima H. Ohno H. (2004). Highly ion conductive flexible films composed of network polymers based on polymerizable ionic liquids. Polymer 45, 1577–1582. 10.1016/j.polymer.2004.01.003

[B67] Wei R. Guo J. Jin L. He C. Xie Y. Zhang X. (2020). Vapor induced phase separation towards anion-/near-infrared-responsive pore channels for switchable anti-fouling membranes. J. Mat. Chem. 8, 8934–8948. 10.1039/d0ta02154g

[B68] Wei R. Yang B. He C. Jin L. Zhang X. Zhao C. (2022). Versatile and robust poly(ionic liquid) coatings with intelligent superhydrophilicity/superhydrophobicity switch in high-efficient oil-water separation. Sep. Purif. Technol. 282, 120100–120113. 10.1016/j.seppur.2021.120100

[B69] Wei R. Yang F. Gu R. Liu Q. Zhou J. Zhang X. (2018). Design of robust thermal and anion dual-responsive membranes with switchable response temperature. ACS Appl. Mat. Interfaces 10, 36443–36455. 10.1021/acsami.8b12887 30277384

[B70] Wu X. Surendran A. Moser M. Chen S. Muhammad B. T. Maria I. P. (2020). Universal spray-deposition process for scalable, high-performance, and stable organic electrochemical transistors. ACS Appl. Mat. Interfaces 12, 20757–20764. 10.1021/acsami.0c04776 32281363

[B71] Xu L. Huang Z. Deng Z. Du Z. Sun T. L. Guo Z. H. (2021). A transparent, highly stretchable, solvent-resistant, recyclable multifunctional ionogel with underwater self-healing and adhesion for reliable strain sensors. Adv. Mat. 33, e2105306. 10.1002/adma.202105306 34647370

[B72] Yang W. He X. Gao J. Guo H. He X. Wan F. (2010). Synthesis, characterization, and tunable wettability of poly(ionic liquid) brushes via nitroxide-mediated radical polymerization (NMP). Chin. Sci. Bull. 55, 3562–3568. 10.1007/s11434-010-3288-z

[B73] Yang Z. Huang R. Zheng B. Guo W. Li C. He W. (2021). Highly stretchable, adhesive, biocompatible, and antibacterial hydrogel dressings for wound healing. Adv. Sci. 8, 2003627–2003638. 10.1002/advs.202003627 PMC8061386 33898178

[B74] Yao X. Zhang S. Qian L. Wei N. Nica V. Coseri S. (2022). Super stretchable, self‐healing, adhesive ionic conductive hydrogels based on tailor‐made ionic liquid for high‐performance strain sensors. Adv. Funct. Mat. 32, 2204565. 10.1002/adfm.202204565

[B75] Ye L. Chen F. Liu J. Gao A. Kircher G. Liu W. (2019). Responsive ionogel surface with renewable antibiofouling properties. Macromol. Rapid Commun. 40, e1900395. 10.1002/marc.201900395 31507007

[B76] Ye Q. Gao T. Wan F. Yu B. Pei X. Zhou F. (2012). Grafting poly(ionic liquid) brushes for anti-bacterial and anti-biofouling applications. J. Mat. Chem. 22, 13123–13131. 10.1039/c2jm31527k

[B77] Yu Y. Yang Z. Ren S. Gao Y. Zheng L. (2020). Multifunctional hydrogel based on ionic liquid with antibacterial performance. J. Mol. Liq. 299, 112185–112192. 10.1016/j.molliq.2019.112185

[B78] Yu Z. Wu P. (2021). Underwater communication and optical camouflage ionogels. Adv. Mat. 33, e2008479. 10.1002/adma.202008479 33955597

[B79] Zgrzeba A. Andrzejewska E. Marcinkowska A. (2015). Ionic liquid-containing ionogels by thiol–ene photopolymerization. Kinetics and solvent effect. RSC Adv. 5, 100354–100361. 10.1039/c5ra21254e

[B80] Zhang J. Chen Z. Zhang Y. Dong S. Chen Y. Zhang S. (2021). Poly(ionic liquid)s containing alkoxy chains and bis(trifluoromethanesulfonyl)imide anions as highly adhesive materials. Adv. Mat. 33, e2100962. 10.1002/adma.202100962 34117661

[B81] Zhang J. Shen B. Chen L. Chen L. Mo J. Feng J. (2019). Antibacterial and antifouling hybrid ionic-covalent hydrogels with tunable mechanical properties. ACS Appl. Mat. Interfaces 11, 31594–31604. 10.1021/acsami.9b08870 31407568

[B82] Zhang J. Zhang W. Guo J. Yuan C. Yan F. (2015). Ultrahigh ionic liquid content supramolecular ionogels for quasi-solid-state dye sensitized solar cells. Electrochim. Acta 165, 98–104. 10.1016/j.electacta.2015.02.244

[B83] Zhang X. Chen Q. Wei R. Jin L. He C. Zhao W. (2020). Design of poly ionic liquids modified cotton fabric with ion species-triggered bidirectional oil-water separation performance. J. Hazard. Mat. 400, 123163–123178. 10.1016/j.jhazmat.2020.123163 32569985

[B84] Zhang Y. Yuan B. Zhang Y. Cao Q. Yang C. Li Y. (2020). Biomimetic lignin/poly(ionic liquids) composite hydrogel dressing with excellent mechanical strength, self-healing properties, and reusability. Chem. Eng. J. 400, 125984–125993. 10.1016/j.cej.2020.125984

[B86] Zhao W. Ye Q. Hu H. Wang X. Zhou F. (2015). Fabrication of binary components based on a poly(ionic liquid) through “grafting” and “clicking” and their synergistic antifouling activity. RSC Adv. 5, 100347–100353. 10.1039/c5ra23391g

[B87] Zhao W. Ye Q. Hu H. Wang X. Zhou F. (2014). Grafting zwitterionic polymer brushes via electrochemical surface-initiated atomic-transfer radical polymerization for anti-fouling applications. J. Mat. Chem. B 2, 5352–5357. 10.1039/c4tb00816b 32261755

[B88] Zheng L. Li J. Yu M. Jia W. Duan S. Cao D. (2020). Molecular sizes and antibacterial performance relationships of flexible ionic liquid derivatives. J. Am. Chem. Soc. 142, 20257–20269. 10.1021/jacs.0c10771 33179921

[B89] Zhuo Y. Xiao S. Håkonsen V. He J. Zhang Z. (2020). Anti-icing ionogel surfaces: Inhibiting ice nucleation, growth, and adhesion. ACS Mat. Lett. 2, 616–623. 10.1021/acsmaterialslett.0c00094

